# Inflammatory Biomarkers Predicting Major Adverse Cardiovascular Events in People Living With HIV: A Systematic Review and Meta‐Analysis

**DOI:** 10.1002/jia2.70101

**Published:** 2026-04-27

**Authors:** Ashley Murray, Niamh Louwman, Augustine Luk, Andrew Parker, Nomalanga Madyiwa, Harold Parker, Kathryn Woodward, Carola Fajardo Gallegos, Jienchi Dorward, Richard J. Stevens, Thomas R. Fanshawe

**Affiliations:** ^1^ Nuffield Department of Primary Care Health Sciences University of Oxford Oxford UK; ^2^ Medical Sciences Division University of Oxford Oxford UK; ^3^ Nuffield Department of Medicine University of Oxford Oxford UK; ^4^ Oxford University Hospitals NHS Foundation Trust John Radcliffe Hospital Oxford UK; ^5^ Perinatal HIV Research Unit University of the Witwatersrand Johannesburg South Africa; ^6^ Nuffield Department of Population Health University of Oxford Oxford UK; ^7^ School of Medicine and Dentistry Griffith University Gold Coast Australia; ^8^ School of Medicine La Salle University Mexico City Mexico; ^9^ Centre For the AIDS Programme of Research in South Africa (CAPRISA) University of KwaZulu‐Natal Durban South Africa

**Keywords:** Biomarkers, Cardiovascular Diseases, HIV, Inflammation, Prognosis, Systematic Review

## Abstract

**Introduction:**

Chronic inflammation is a unique contributor to cardiovascular disease (CVD) risk among people living with HIV, yet there is a lack of consensus on the predictive utility of inflammatory biomarkers in this population. We conducted a systematic review assessing the predictive value of inflammatory biomarkers for major adverse cardiovascular events in people living with HIV to inform their potential integration into CVD risk assessment.

**Methods:**

MEDLINE, Embase and Google Scholar were searched for articles published up to 01 May 2024. We included prospective cohort and nested case‐control studies of adults living with HIV with inflammatory biomarker measurements in blood and at least one year of follow‐up to major adverse cardiovascular events. Risk of bias was assessed using the Quality in Prognostic Studies (QUIPS) tool. Where at least two studies reported the same type of effect measure for a biomarker, results were pooled using an inverse variance heterogeneity model.

**Results:**

Among 5156 screened citations, 21 studies reporting 31 inflammatory biomarkers met inclusion criteria. Meta‐analysis showed high‐sensitivity C‐reactive protein (hsCRP) positively associated with future cardiovascular events (hazard ratio =  1.86 per log_10_ unit; 95% CI 1.39–2.50, *n* = 5,254). Three biomarkers, interleukin 6 (IL‐6), D‐dimer, and N‐terminal pro‐brain natriuretic peptide (NT‐proBNP), demonstrated positive, statistically significant associations with adverse cardiovascular outcomes in at least two non‐overlapping studies, though heterogeneous effect measures precluded meta‐analysis. Most research (14/21 studies) was conducted exclusively in high‐income settings, and female representation was low (median proportion = 15.5%; IQR 8.4–20.9%). All but three studies had a moderate or high risk of bias in at least one domain.

**Discussion:**

We identified several inflammatory biomarkers with potential prognostic value, but most associations were derived from single or heterogeneous studies. The certainty of evidence is reduced by methodological heterogeneity, few high‐quality studies and the underrepresentation of low‐ and middle‐income countries (LMICs).

**Conclusions:**

Consistent positive associations between inflammatory biomarkers and future CVD in people living with HIV support a central role of inflammation in HIV‐related CVD. Representative, large‐scale studies that include women and LMICs are needed to guide the integration of candidate biomarkers into CVD risk prediction models.

**PROSPERO Number:**

CRD42024542944

**Abbreviations**
ACC: American College of Cardiology
aHR: Adjusted Hazard Ratio
AIDS: Acquired Immunodeficiency Syndrome
aOR: Adjusted Odds Ratio
ART: Antiretroviral Therapy
BNP: B‐type Natriuretic Peptide
CC: Case‐Control
CCL2: Chemokine Ligand 2
CCL25: Chemokine Ligand 25
CIMT: Carotid Intima‐Media Thickness
COH: Cohort Study
CRP: C‐Reactive Protein
CVD: Cardiovascular Disease
Gal‐9: Galectin‐9
GM‐CSF: Granulocyte‐Macrophage Colony‐Stimulating Factor
HIV: Human Immunodeficiency Virus
HR: Hazard Ratio
hsCRP: High‐Sensitivity C‐Reactive Protein
hsTNT: High‐Sensitivity Troponin T
ICAM‐1: Intercellular Adhesion Molecule 1
IFN‐γ: Interferon Gamma
IL: Interleukin
IL‐1R1: Interleukin‐1 Receptor 1
IL‐1Ra: Interleukin‐1 Receptor Antagonist
IP‐10: Interferon Gamma‐Induced Protein 10
IQR: Interquartile Range
ITGA11: Integrin Subunit Alpha 11
IVhet: Inverse Variance Heterogeneity
LDL: Low‐Density Lipoprotein
LMIC: Low‐ And Middle‐Income Country
MACE: Major Adverse Cardiovascular Events
MPO: Myeloperoxidase
NPX: Normalised Protein eXpression
NT‐proBNP: N‐terminal Pro B‐type Natriuretic Peptide
OR: Odds Ratio
oxLDL: Oxidised Low‐Density Lipoprotein
PAI‐1: Plasminogen Activator Inhibitor‐1
PICOTS: Population, Index prognostic factor, Comparator prognostic factors, Outcome, Timing, Setting
PLA2G7: Phospholipase A2 Group VII
PLWH: People Living with HIV
PRISMA: Preferred Reporting Items for Systematic Reviews and Meta‐Analyses
PROSPERO: International Prospective Register of Systematic Reviews
QUIPS: Quality in Prognosis Studies
RCT: Randomised Controlled Trial
ROS: Reactive Oxygen Species
sCD: Soluble Cluster of Differentiation
SD: Standard Deviation
sTNFR‐I / sTNFR‐II: Soluble Tumour Necrosis Factor Receptors I and II
TIMP‐1: Tissue Inhibitor of Metalloproteinases‐1
TMAO: Trimethylamine N‐oxide
TNF‐α: Tumour Necrosis Factor Alpha
VCAM‐1: Vascular Cell Adhesion Molecule 1

## 1 Introduction

People living with HIV (PLWH) experience a two‐fold higher risk of cardiovascular disease (CVD) [1]. While both traditional risk factors and HIV‐specific risks contribute [2], chronic inflammation has been posited as a unique contributor towards CVD risk in PLWH [3].

**FIGURE 1:**
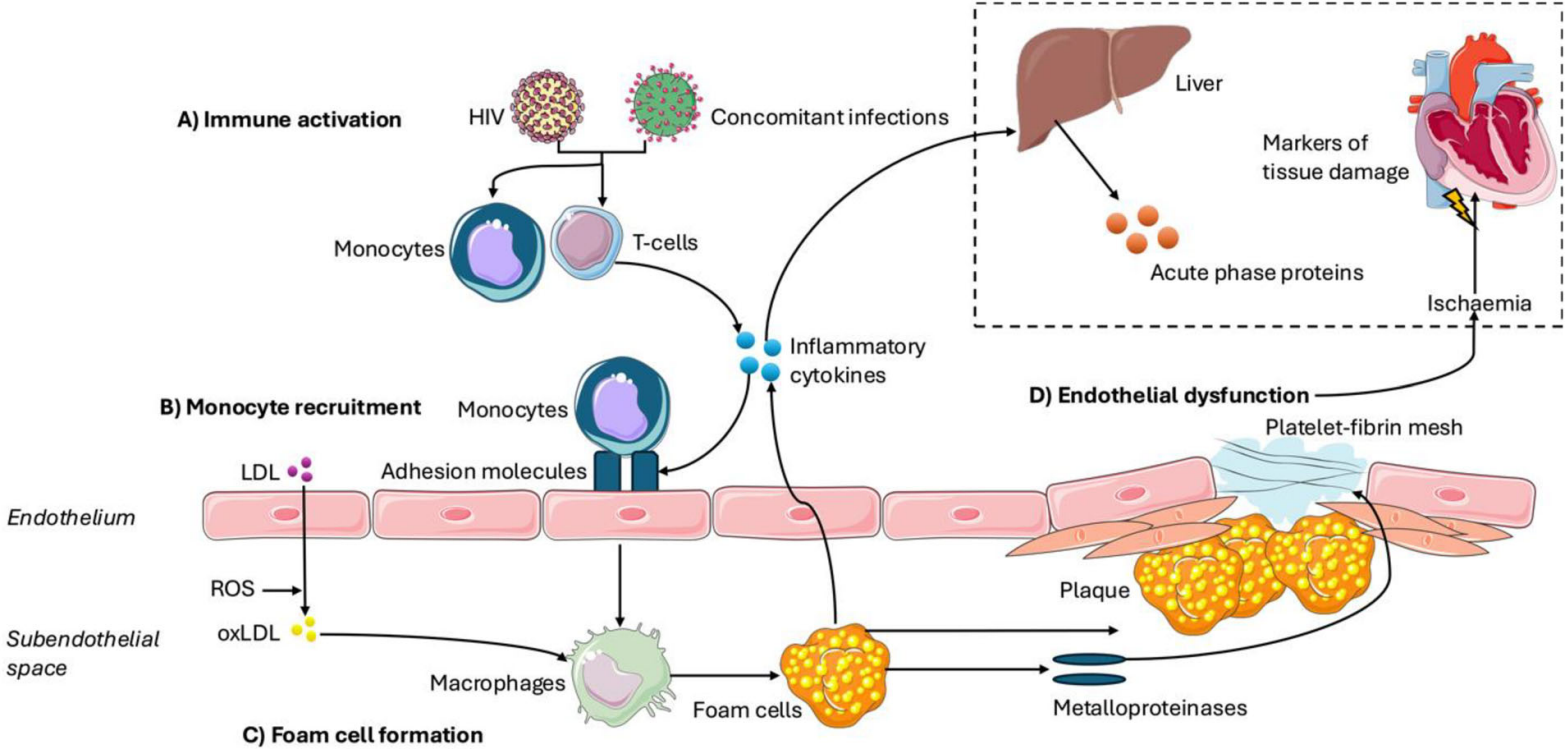
The role of inflammation in progressing atherosclerosis. A) HIV immune activation and concomitant infections lead to T‐cell and monocyte activation. B) Inflammatory cytokines released by activated immune cells increase the expression of adhesion molecules on the endothelium, promoting the recruitment and adhesion of monocytes which differentiate into macrophages. C) Macrophages engulf oxLDL and form foam cells, contributing to plaque formation. D) Chronic inflammation and metalloproteinases weaken the fibrous cap of plaques, increasing the risk of rupture, thrombosis and ischaemia. Systemic inflammation also induces the liver to produce acute‐phase proteins. Images used in this figure were provided by Servier Medical Art (https://smart.servier.com/), licensed under CC BY 4.0 (https://creativecommons.org/licenses/by/4.0/). LDL, Low‐Density Lipoprotein; oxLDL, Oxidised Low‐Density Lipoprotein; ROS, Reactive Oxygen Species.

The HIV virus can cause persistent inflammation and immune cell activation that promotes vascular change [4, 5]. Additionally, co‐infections in PLWH, such as hepatitis, cytomegalovirus and Epstein‐Barr virus, exacerbate ongoing inflammation [6]. Persistent immune activation leads to endothelial dysfunction and vascular plaque formation, resulting in vascular injury [4, 5, 7, 8] (Figure 1).

Measuring inflammatory biomarkers in patients through non‐invasive means offers a promising approach to help quantify this risk. A biomarker is a measurable indicator of biological activity, which can signal normal function, risk of disease, or the body's response to illness [9]. Biomarkers can be detected in body fluids, excretions or tissues and offer valuable insights into physiological processes. Inflammatory biomarkers measured in the serum or plasma can reflect the underlying pathophysiological processes and serve as indicators of vascular injury and immune cell activation [8].

However, existing cardiovascular risk prediction models do not incorporate such inflammatory biomarker information [10]. These models, largely developed using general populations from high‐income settings, demonstrate moderate discriminatory ability (C‐statistic ranging between 0.7‐0.8) when applied in a population of PLWH and frequent miscalibration, reported to typically underestimate cardiovascular risk [11, 12].

Given the growing interest in clinical risk prediction models and their suboptimal performance in PLWH [11, 12, 13], it is worth exploring whether inflammatory biomarkers are a predictive, yet unmeasured, factor in cardiovascular risk in PLWH. However, there remains insufficient consensus on the level of evidence regarding the utility of inflammatory biomarkers in predicting future CVD in PLWH. This systematic review aims to evaluate the prognostic utility of circulating inflammatory biomarkers in predicting major adverse cardiovascular events (MACE) in PLWH to inform future risk stratification in this population.

## 2 Methods

The study methods were drafted in accordance with recommendations outlined by the Cochrane Prognosis Methods Group [14]. The protocol was registered on PROSPERO (CRD42024542944).

### 2.1 Review Question and Eligibility

The review question was developed using the PICOTS (Population, Index prognostic factor, Comparator prognostic factors, Outcome, Timing, Setting) framework [14] (Table 1).

**TABLE 1:** PICOTS^*^ Framework.

Population	Index	Comparator	Outcome	Timing	Setting
Adults living with HIV	Inflammatory biomarkers measured in plasma or serum	N/a	Major adverse cardiovascular events	At least one year of longitudinal data	Any

* Population, Index Prognostic Factor, Comparator Prognostic Factors, Outcome, Timing, Setting.

We included adults (≥ 18 years old) living with HIV across all geographical locations and any healthcare setting, and we excluded studies where the prevalence of known antecedent CVD in the cohort exceeded 80% to ensure applicability to primary prevention populations. Studies including a subset of participants living with HIV were considered eligible if data for this subset was analysed and presented separately. Participants were included both with and without antiretroviral therapy (ART) exposure.

Biomarkers related to systemic inflammation and atherosclerotic inflammation measured in plasma or serum were included. Based on key categories outlined in the literature [8, 15, 16, 17], we considered cytokines, acute phase proteins, adhesion molecules and modulation factors, prothrombotic factors, tissue‐remodelling factors, oxidative stress markers and markers of tissue damage to be eligible. We excluded genetic markers, radiological findings or gastrointestinal microbiome profiling. Studies that measured biomarkers at baseline were considered eligible. Where studies reported biomarkers from multiple time points, we extracted data from the measurement closest to ART initiation, where applicable, given the current era of universal treatment. Details of biomarker collection timepoints are outlined in Supporting Table S1.

Studies were included if their reported endpoint included at least one of the following cardiovascular outcomes, consistent with the components of MACE as outlined by the American College of Cardiology Guidelines [18]: (i) acute myocardial infarction, (ii) acute coronary syndromes, (iii) stable or unstable angina, (iv) coronary or other arterial revascularisation, (v) stroke, (vi) transient ischemic attack, (vii) peripheral arterial disease and (viii) cardiovascular‐related death. We also included heart failure as an outcome, as a chronic and novel immune response in PLWH has been directly implicated in the mechanism of heart failure [19]. Outcomes could be recorded through clinical evaluation, participant self‐report, examination of medical records or billing information.

We included studies that temporally measured biomarkers prior to outcome collection in a longitudinal framework. Prospective studies had to contain at least one year of follow‐up, and for retrospective studies, we required at least a one‐year interval between the biomarker measurement and the occurrence of the outcome event. If the timing was unclear, we contacted the authors for clarification. We included data from longitudinal cohort studies, case‐control studies nested in cohorts and observational segments of randomised controlled trials. We excluded case reports, case series, editorials and research letters without primary data, and cross‐sectional studies. Full published papers were preferred to conference abstracts or unpublished data, where these originated from the same study.

When eligible studies involved overlapping populations, we included all relevant studies in the review, but the analysis was restricted to the largest study to avoid duplicating data.

### 2.2 Search Strategy and Selection Process

MEDLINE and Embase were searched using the search queries without language or publication date restrictions until 01 May 2024. Records were screened for eligibility between May and October 2024. A search query using keywords and controlled vocabulary was developed in consultation with a university librarian (Supporting Information file 1). ProQuest and Google Scholar were used to search for grey literature, and Google Scholar was used for forward citation searching. Duplicate screening of all records was performed by two reviewers (AM and one of NL, AL, AP, NM, HP, KW or CFG), who screened all records independently against the eligibility criteria. Discrepancies were resolved by consensus or consultation with a third senior reviewer (JD, RJS or TRF). No automated tools were used to assess the eligibility of reports.

### 2.3 Data Extraction

A data extraction template followed the modified CHARMS‐PF (Checklist for Critical Appraisal and Data Extraction for Systematic Reviews of Prognostic Factor studies) checklist [14]. Extraction was performed by AM and checked by TRF to ensure consistency. Where results critical for calculating effect estimates were unavailable, the study team contacted the authors for further information. Where confidence intervals were not reported and authors did not respond to requests, the standard error was estimated from the effect estimate and the *p*‐values. To ensure comparability across studies with different log‐transformed biomarker scales, we applied a standardised conversion where feasible (Supporting Information file 1).

The quality of the included studies was evaluated using the QUIPS (Quality in Prognosis Studies) tool [20]. Two reviewers (AM and NL) independently judged the quality of the studies against the QUIPS criteria. Disagreements were resolved through consultation with a third senior reviewer (JD, RJS or TRF). We presented our results as a domain‐level summary, as it is recommended against summing individual domains into an overall score [20]. As the QUIPS domain relating to study attrition is poorly applicable to case‐control studies, after discussion with the authors of the QUIPS tool, we marked this domain as ‘not applicable’ for those studies due to study design rather than methodological weakness. We considered the potential for selective reporting bias by examining whether studies reported results for all measured biomarkers or only a subset.

### 2.4 Data Analysis and Narrative Synthesis

If the same biomarker was reported with the same type of effect estimate in at least two cohorts or studies, then a meta‐analysis was performed. As substantial heterogeneity is expected in prognostic studies [21], hazard ratios (HRs) were pooled together using the inverse variance heterogeneity (IVhet) model to account for between‐study heterogeneity and produce conservative confidence interval estimates, particularly for smaller studies [22].

While we pre‐specified quantifying heterogeneity, guidelines note that these measures have limited power to detect heterogeneity when there are few studies [23]. We also planned to carry out a sensitivity analysis using the largest studies and a subgroup analysis excluding studies with a high risk of bias, but there were insufficient studies to conduct these preplanned analyses.

When effect estimates were reported differentially across studies (odds ratios and hazard ratios), we did not pool these and instead provided a narrative synthesis. Where multiple estimates were reported within a study, we prioritised adjusted over unadjusted results and continuous measures over binary or categorical outcomes. Similarly, where multiple adjusted models were reported, we prioritised the most fully adjusted model that did not include other inflammatory biomarkers. We assessed whether reported associations were statistically significant, defined as *p* < 0.05 or confidence intervals excluding the null value. Furthermore, we assessed the overall strength of evidence underpinning our conclusions for each biomarker in terms of the risk of bias of studies, whether studies reported all planned analyses, the variability of estimates and the width of confidence intervals for effect estimates across studies.

Data analysis was performed using R Statistical Software version 4.4.1 [24]. Meta‐analysis using the IVhet method was carried out using the ‘admetan’ package in Stata 18 [25, 26], and the visualisation of risk of bias was carried out using the ‘robvis’ package in R [27].

Ethical approval and participant consent were not required for this systematic review, as it involved analysis of publicly available and previously published evidence.

## 3 Results

### 3.1 Study Selection & Characteristics

**FIGURE 2:**
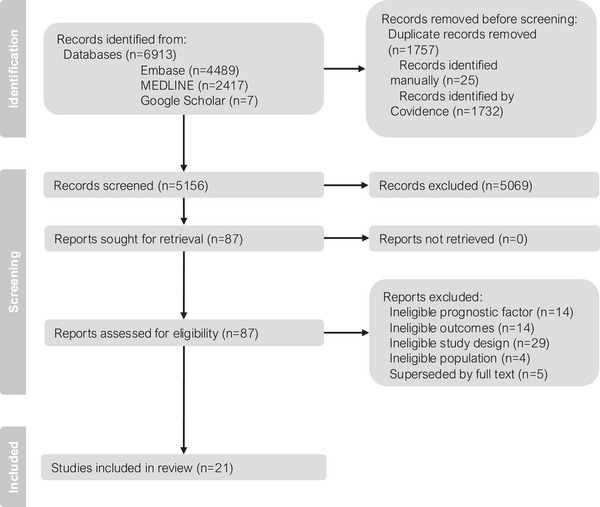
PRISMA flow diagram. Adapted from Page et al. [28]. Licensed under CC BY 4.0 (https://creativecommons.org/licenses/by/4.0/).

The search strategy yielded 6913 citations, of which 21 articles were included in the review (Figure 2).

Table 2 outlines the characteristics of the included studies. Extended characteristics of the studies are outlined in Supporting Table S1.

**TABLE 2:** Characteristics of included studies.

			Case‐control (CC) or cohort (COH)	Eligible biomarker(s) reported			
Authors	Year	Location		**D‐dimer**	**hsCRP**	**IL‐6**	**Other**
**Danish HIV Cohort Study**							
Knudsen et al. [29]	2014	Denmark^a^	CC		✓		PAI‐1
Knudsen et al. [30]	2013	Denmark^a^	CC				sCD163
Haissman et al. [31]	2016	Denmark^a^	CC				TMAO
Hoel et al. [32]	2020	Denmark^a^	CC				IL‐1Ra, IL‐1R1
Rasmussen et al. [33]	2016	Denmark^a^	CC				suPAR
**AIDS Clinical Trials Group Longitudinal Linked Randomized Trials**							
Tenorio et al. [34]	2014	USA^b^	CC	✓		✓	IP‐10, sTNFR‐I, sTNFR‐II, sCD14
Premeaux et al. [35]	2021	USA^b^	CC				Gal‐9
**Strategies for Management of Antiretroviral Therapy Study**							
Borges et al. [36]	2016	Global^c^	COH	✓	✓	✓	
Duprez et al. [37]	2011	Global^c^	CC				NT‐proBNP
Duprez et al. [38]	2012	Global^c^	COH	✓	✓	✓	
Duprez et al. [39]	2014	Global^c^	CC				Glyc A
Safo et al. [40]	2023	Global^c^	CC			✓	ITGA11, CCL25, PLA2G7
Grund et al. [41]	2016	Global^c^	COH	✓	✓	✓	
Bernardino et al. [42]	2023	Spain	CC				sCD14, sCD163, CCL2
de Leuw et al. [43]	2021	Germany	COH		✓		hsTNT, NT‐proBNP
Marconi et al. [44]	2018	USA	COH				Total bilirubin
Mocumbi et al. [45]	2020	Mozambique	COH	✓			
Ford et al. [46]	2010	USA	CC	✓	✓	✓	VCAM‐1, ICAM‐1, TIMP‐1, MPO, IL‐2, IL‐10, sCD14, TNF‐α, TNF‐γ
Missailidis et al. [47]	2018	Sweden	COH				TMAO
Reinsch et al. [48]	2019	Germany	COH				BNP
Kirkegaard‐Klitbo et al. [49]	2017	Denmark	COH				sCD163

Abbreviations: BNP, B‐type Natriuretic Peptide; CC, Case‐Control; CCL2, Chemokine Ligand 2; CCL25, Chemokine Ligand 25; COH, Cohort Study; CRP/hsCRP, (High‐Sensitivity) C‐Reactive Protein; Gal‐9, Galectin‐9; GM‐CSF, Granulocyte‐Macrophage Colony‐Stimulating Factor; ICAM‐1, Intercellular Adhesion Molecule 1; IFN‐γ, Interferon Gamma; IL, Interleukin; IL‐1Ra, IL‐1 Receptor Antagonist; IL‐1R1, IL‐1 Receptor 1; IP‐10, Interferon Gamma‐Induced Protein 10; ITGA11, Integrin Subunit Alpha 11; MPO, Myeloperoxidase; NT‐proBNP, N‐terminal Pro B‐type Natriuretic Peptide; PAI‐1, Plasminogen Activator Inhibitor‐1; PLA2G7, Phospholipase A2 Group VII.; sCD, Soluble Cluster of Differentiation; sTNFR‐I/sTNFR‐II, Soluble TNF Receptors I and II; suPAR, Soluble Urokinase‐Type Plasminogen Activator Receptor; TIMP‐1, Tissue Inhibitor of Metalloproteinases‐1; hsTNT, (High Sensitivity) Troponin T; TNF‐α, Tumor Necrosis Factor Alpha; TMAO, Trimethylamine N‐oxide; VCAM‐1, Vascular Cell Adhesion Molecule 1.

^a^ Overlapping cohort (Danish HIV Cohort Study).

^b^ Overlapping cohort (AIDS Clinical Trials Group Longitudinal Linked Randomized Trials).

^c^ Overlapping cohort (Strategies for Management of Antiretroviral Therapy Study). Conducted across Africa, Asia, Australia, Europe, North America, South America.

Of the 21 studies, 12 were case‐control studies nested within larger cohorts [29, 30, 31, 32, 33, 34, 35, 37, 39, 40, 42, 46]. One study was a conference abstract [39]. Fourteen of the twenty‐one studies were conducted exclusively in high‐income settings. Among studies that comprehensively reported participant ethnicity, the median proportion of White participants was 81.0% (IQR: 70.9–90.2%, range: 78.2%–100%). Female representation was low, with a median proportion of 15.5% (IQR: 8.4–20.9, range: 1.9–58.6%). From the studies that reported smoking status as a cardiovascular risk factor, the median proportion of participants who were current or past smokers was 66.0% (IQR: 38.8–87.0%, range: 4.2–96.6%) (Supporting Table S1).

### 3.2 Study Results

We identified 31 eligible inflammatory biomarkers reported across these eligible studies. hsCRP (high‐sensitivity C‐reactive protein), D‐dimer and IL‐6 (interleukin 6) were the biomarkers most frequently reported. Of all eligible biomarkers reported, seven biomarkers were reported with effect estimates in at least two studies, namely hsCRP [29, 36, 38, 41, 43], IL‐6 [34, 36, 38, 40, 41], D‐dimer [34, 36, 38, 41], NT‐proBNP (N‐terminal pro‐brain natriuretic peptide) [37, 43], TMAO (Trimethylamine N‐oxide) [31, 47], sCD163 (Soluble Cluster of Differentiation 163) [42, 49] and sCD14 (Soluble Cluster of Differentiation 14) [34, 42]. Of the seven biomarkers reported with effect estimates in at least two studies, the issue of overlapping cohorts applied only to the SMART cohort in relation to hsCRP, IL‐6 and D‐dimer [36, 38, 41]. In this case, we retained the estimate from Duprez et al. [38], as it included the largest (n = 5098) analysis of the SMART cohort. Table 3 summarises the effect measures for these seven biomarkers.

**TABLE 3:** Biomarkers with evidence summarised across multiple studies.

Biomarker	Study	Association (95% CI)	Comparison
hsCRP	de Leuw et al., 2021	HR = 1.22 (0.53–2.82)	Per log_10_
	Duprez et al., 2012	aHR = 1.94 (1.49–2.50)	Per log_10_ ^a^
	Knudsen et al., 2014	OR = 8.5 (1.1–67.5)	>3 vs ≤3 mg/L
IL‐6	Duprez et al., 2012	aHR = 2.63(1.93–3.56)	Per log_10_ ^a^
	Safo et al., 2023	aOR 1.54 (1.18–2.00)	Per 1 SD in NPX
	Tenorio et al., 2014^b^	aOR = 2.8 (0.7–10.5)	Per log_10_
D‐dimer	Duprez et al., 2012	aHR = 2.23(1.57–3.11)	Per log_10_ ^a^
	Tenorio et al., 2014^b^	aOR = 6.2 (1.1–35.6)	Per log_10_
NT‐proBNP	de Leuw et al., 2021	HR = 2.61 (1.42–4.80)	Per log_10_
	Duprez et al., 2011	aOR = 1.16(1.06–1.27)	Per log_10_ ^c^
TMAO	Haissman et al., 2016^b^	OR = 1.6 (0.404–6.327)^d^	>5 or ≤ 5
	Missailidis et al., 2018	HR = 2.76 (0.29–26.70)	>4.93 or ≤ 4.93
sCD163	Bernardino et al., 2023	aOR = 1.0 (1.0–1.0)	Per pg/mL
	Kirkegaard‐Klitbo et al., 2017	aHR = 1.04 (0.97–1.12)	Per mg/l
	Knudsen et al., 2013	OR = 1.05 (0.85–1.29)	Per log_10_
sCD14	Bernardino et al., 2023	aOR = 1.0 (1.0–1.0)	Per pg/mL
	Tenorio et al., 2014^b^	aOR = 9.0 (0.2 –339)	Per log_10_

Abbreviations: aHR, Adjusted Hazard Ratio; aOR, Adjusted Odds Ratio; CI, Confidence Interval; HR, Hazard Ratio; hsCRP, (High‐Sensitivity) C‐Reactive Protein; IL‐6, Interleukin 6; NPX, Normalised Protein eXpression; NT‐proBNP, N‐terminal Pro B‐type Natriuretic Peptide; OR, Odds Ratio; sCD, Soluble Cluster of Differentiation; SD, Standard Deviation; TMAO, Trimethylamine N‐oxide.

^a^ Effect sizes were originally reported per 1 SD increase in log_10_‐transformed hsCRP. This was converted to per‐log_10_ using the reported SD of hsCRP in the study population.

^b^ Effect sizes are based on post‐antiretroviral therapy baseline measurements for comparability between cohorts.

^c^ Effect sizes were originally reported per log_e_. This was converted to per‐log_10_ to be comparable.

^d^ Confidence intervals were calculated from *p*‐value and effect estimate.

hsCRP, IL‐6, D‐dimer, and NT‐proBNP were each associated with increased risk of adverse outcomes in at least one study [29, 34, 38, 43]. Odds ratio estimates from Tenorio et al. [34] for both IL‐6 and D‐dimer had limited precision due to wide confidence intervals. Studies of TMAO, sCD163 and sCD14 generally reported effect estimates with wide confidence intervals and confidence intervals that crossed the null [30, 31, 34, 47, 49]. hsCRP was the only biomarker reported by at least two non‐overlapping studies using the same measure of effect where conversion of scales was possible. The pooled hazard ratio for hsCRP was 1.86 (95% CI: 1.39‐2.50) per log_10_ unit (Supporting Figure S1). Biomarkers reported by only a single study are summarised separately (Supporting Table S2).

### 3.3 Risk of Bias

**FIGURE 3:**
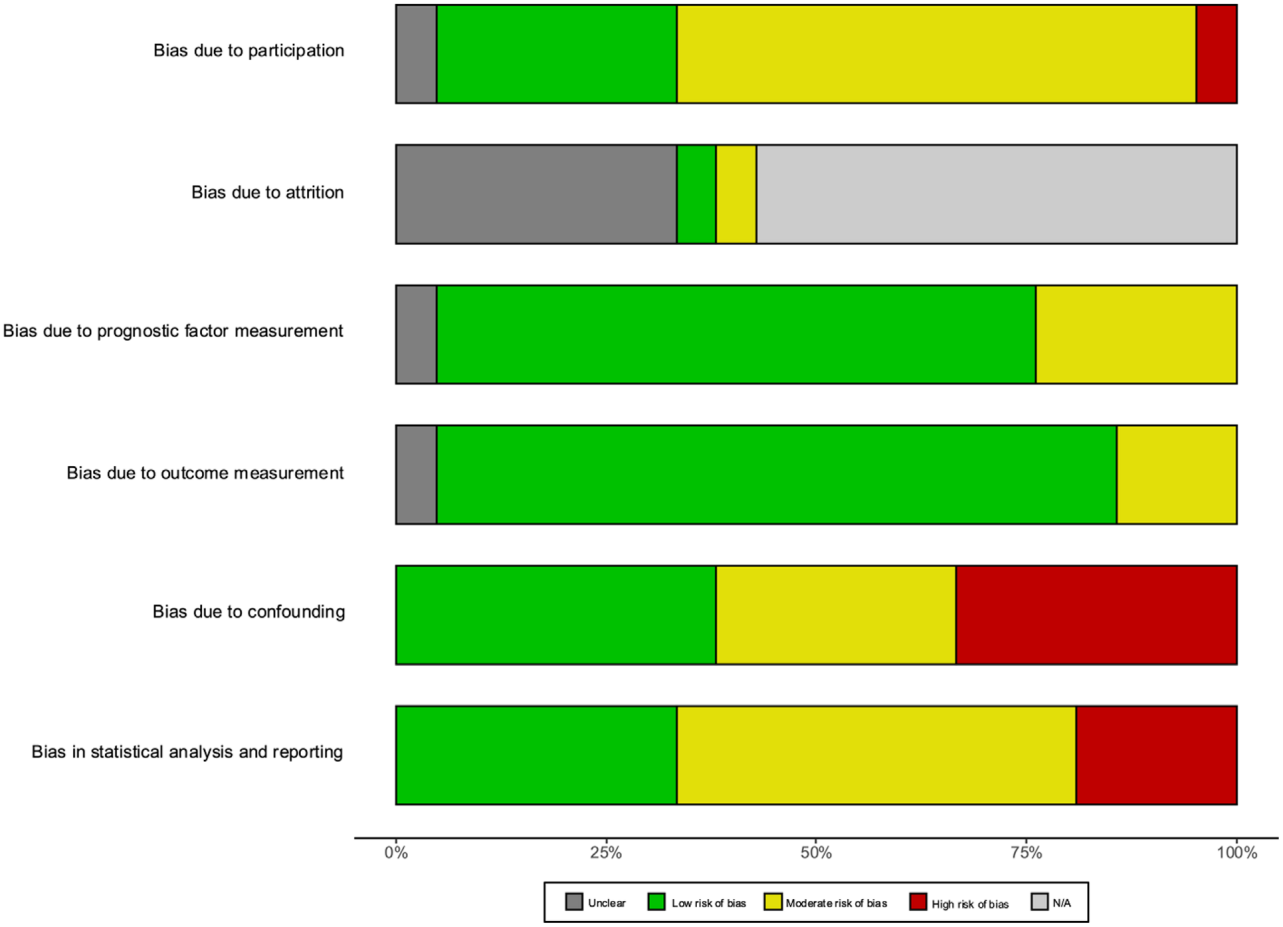
Summary of risk of bias assessment across QUIPS domains.

Study quality varied considerably (Figure 3). All but three studies scored a moderate or high risk of bias in at least one domain (Supporting Figure S2). For study participation, 13 studies were rated at moderate risk of bias [29, 30, 32, 33, 34, 35, 36, 37, 40, 42, 45, 46, 47], one study had a high risk of selection bias [31], while six were low risk [38, 41, 43, 44, 48, 49]. Study attrition was generally unclear for most cohort studies except for two studies [43, 45], and the domain was not applicable for case‐control studies. Studies generally performed well in the domains of prognostic factor and outcome measurement, with most demonstrating a low risk of bias for prognostic factor measurement [30, 32, 33, 34, 35, 36, 37, 38, 40, 42, 43, 44, 45, 48, 49] and outcome measurement [29, 30, 31, 32, 33, 34, 35, 36, 37, 38, 40, 41, 43, 44, 47, 48, 49]. The domains at higher risk of bias were those related to confounding and statistical analysis and reporting. There was moderate risk of bias related to confounding in six studies [29, 30, 32, 33, 46, 49], and seven studies at a high risk of bias either due to no or inadequate adjustment for confounders [31, 35, 41, 42, 43, 45, 47]. Ten studies were at a moderate risk of statistical analysis and reporting bias [30, 33, 35, 39, 40, 41, 42, 43, 46], and four were at a high risk of bias, three of which were largely due to selective reporting of biomarker results [29, 31, 32].

When considering the risk of bias alongside reported biomarkers with evidence synthesised across multiple studies, studies contributing data for IL‐6 and D‐dimer were not assessed to be at high risk of bias across most domains [34, 38, 40]. For hsCRP, which was the only biomarker eligible for meta‐analysis, one of the studies meta‐analysed did not raise major concerns [38], while the other had a high risk of bias due to confounding [43]. A third study reporting odds ratios on a categorical scale for hsCRP was excluded from the meta‐analysis and had a high risk of bias due to selective reporting of results [29]. Similarly, for NT‐proBNP, while one study generally had a low risk of bias across domains [37], the other was found to have inadequate control of confounding [43]. For studies reporting TMAO, sCD163 and sCD14, there was generally a high risk of bias due to confounding [31, 42, 47] and selective reporting [31].

Most studies did not report or account for ART interruptions or adherence. Among the SMART trial studies, some used participants from both randomisation arms and adjusted for or matched on treatment assignment [37, 38, 40], while others restricted analyses to the continuous ART arm only [36, 41]. However, adjusting for randomised treatment assignment is not equivalent to accounting for real‐world ART interruptions or variable adherence during follow‐up, which no studies directly addressed.

## 4 Discussion

This systematic review identified 31 inflammatory biomarkers and investigated the predictive utility of seven biomarkers with reported effect sizes across multiple studies for major adverse cardiovascular events in people living with HIV. Among the biomarkers reported, hsCRP, IL‐6 and D‐dimer were the most frequently studied. hsCRP, IL‐6, D‐dimer and NT‐proBNP demonstrated positive, statistically significant associations with major adverse cardiovascular outcomes in at least two non‐overlapping studies. However, we were only able to conduct a meta‐analysis of two studies that contributed data towards hsCRP due to methodological heterogeneity across studies. The pooled result and average direction of effect for hsCRP indicated a significant, positive association with future major adverse cardiovascular outcomes. However, given the limited number of studies contributing to this analysis, these findings should be interpreted with caution.

While hsCRP, IL‐6, D‐dimer and NT‐proBNP were associated with adverse cardiovascular outcomes in at least two studies each, the strength of this evidence varied. Studies contributing data on IL‐6 and D‐dimer were generally at lower risk of bias [34, 38, 40]. Of the three studies without a moderate or high risk of bias across domains, one of them [38] contributed pooled data towards the hsCRP meta‐analysis, potentially strengthening confidence in the association between hsCRP and MACE despite the limited number of studies. Meanwhile, the evidence for NT‐proBNP came from studies of mixed methodological quality [36, 42]. In contrast, the certainty of evidence supporting associations between TMAO, sCD163 and sCD14 with MACE was low. Across the included studies reporting these biomarkers, confidence intervals were wide, and no statistically significant associations were observed. Moreover, the majority of these studies were at high risk of bias, particularly due to inadequate adjustment for confounding variables and selective reporting [31, 42, 47]. These limitations temper confidence in the observed associations.

To our knowledge, there is only one previous systematic review examining the relationship between inflammatory biomarkers and cardiovascular outcomes in PLWH in relation to clinically manifest CVD [50]. Their findings included eight studies contributing data on clinical CVD outcomes and suggested that C‐reactive protein (CRP), IL‐6 and D‐dimer appeared to be associated with increased CVD risk, which is in keeping with our results. The authors also examined carotid intima‐media thickness (CIMT) as a surrogate marker for CVD, yet most included studies were cross‐sectional by design, making it difficult to establish causality or compare effect estimates directly. Of the studies included in the aforementioned review, our review also identified Ford et al. [46], Knudsen et al. [30], Tenorio et al. [34] and Duprez et al. [38], with our review additionally including a broader and more recent evidence base of 21 studies. Our review differs by focusing exclusively on studies that reported major adverse cardiovascular events only, as our focus was on clinical outcomes rather than surrogate or proxy endpoints. Furthermore, we excluded cross‐sectional studies entirely in order to focus on the prognostic function of biomarkers, as cross‐sectional designs do not establish temporality and limit inferences about the directionality of associations with clinical outcomes.

Our findings associating specific biomarkers to cardiovascular outcomes are consistent with the chronic immune activation and inflammation associated with HIV acquisition, supporting their biological plausibility. Macrophage activation is commonly marked by elevated levels of IL‐6, sCD14 and sCD163, while IL‐6 itself can additionally stimulate the release of acute phase reactant proteins while simultaneously driving endothelial dysfunction and coagulation [51, 52]. CRP is produced in response to IL‐6 and directly promotes atherogenesis by inducing endothelial adhesion molecule expression and enhancing monocyte recruitment into the vascular intima [53]. D‐dimer, a marker of fibrin degradation, reflects ongoing activation of coagulation and fibrinolysis and is elevated in atherosclerosis, reflecting ongoing clot formation and breakdown associated with plaque inflammation, rupture and thrombosis [54]. NT‐proBNP is secreted in response to increases in myocardial wall stress, vascular stiffness and afterload on the heart, and atherosclerotic processes could also plausibly drive its release [55, 56].

In studies of the general population, fibrinogen, IL‐6, galectin‐3 and CRP have been associated with the risk of CVD outcomes [57]. However, while biologically plausible and associated with outcomes, real‐world application with clinical risk prediction scores remains uncertain. Shah et al. found that, while elevated CRP levels were consistently associated with increased coronary heart disease, the discriminative ability of the biomarker was modest both on its own and when added to the Framingham Risk Score in the general population [58]. However, the pathogenesis of CVD in PLWH may differ given the prominent role of chronic immune activation [4]. Traditional risk scores, which do not incorporate markers of immune activation or inflammation, may therefore incompletely capture an important pathogenic mechanism in PLWH.

Moreover, inflammatory processes are likely different in PLWH, potentially influencing biomarker expression, interpretation and clinical relevance. Ashuro et al. emphasise the challenge of generalising biomarker utility, demonstrating variability in treatment responses across different profiles of PLWH [59]. The authors investigated the effect of rosuvastatin therapy on hsCRP, IL‐6 and D‐dimer levels in PLWH and found that rosuvastatin significantly reduced IL‐6 levels, while changes in hsCRP and D‐dimer were not statistically significant. While these results highlight the potential for targeted interventions to modify inflammatory pathways implicated in HIV‐associated CVD, biomarker levels and responses varied based on individual characteristics such as age, antiretroviral therapy duration and baseline immune status, complicating the clinical utility of biomarker‐based risk stratification in PLWH.

This study had several notable strengths. We employed a comprehensive search strategy and searched multiple databases, reducing the risk of selection bias. There were no time or language restrictions, and we included grey literature to thoroughly assess the current state of the literature and minimise publication bias. We excluded cross‐sectional studies, as they do not allow for the assessment of temporal relationships between biomarker levels and future major adverse cardiovascular disease risk, thereby strengthening the validity of the conclusions drawn.

Careful handling of missing data was undertaken by reaching out to study authors, estimating standard errors where necessary and standardising effect estimates across different log‐transformed biomarker scales. Furthermore, the decision to restrict the analysis to the largest dataset prevented duplication of results and inflating associations.

Despite the strengths of our approach, several limitations should be considered when interpreting our findings. Our comprehensive search meant that we included a range of study designs and biomarker measurement protocols, which introduced heterogeneity, making direct comparisons challenging. For retrospective studies, we required at least a one‐year interval between biomarker measurement and the occurrence of the outcome event to ensure appropriate temporality between exposure and outcome, but this may have excluded studies with shorter follow‐up times. Furthermore, while we followed guidance per Cochrane recommendations, the QUIPS tool was not fully applicable to case‐control studies and required modifications.

Almost all included studies exhibited a moderate or high risk of bias in at least one domain. The domains most frequently at risk were those related to confounding and statistical analysis and reporting, with the latter often reflecting selective reporting of biomarker‐outcome associations. Selective reporting bias was evident in several studies, where results were presented only for a subset of measured biomarkers results [29, 31, 32]. These reporting limitations are particularly important in prognostic research, where multiple biomarkers may be explored but only a few are presented based on post hoc findings [60]. The absence of covariate adjustment or the failure to include CVD risk factors further limited interpretability [31, 35, 41, 42, 43, 45, 47].

Two studies enrolling participants diagnosed with heart failure were excluded [61, 62], and included studies had a low or unreported prevalence of prior CVD. Consequently, our findings may not be generalisable to secondary prevention settings or PLWH with established cardiovascular disease. Furthermore, most included studies did not report how ART interruptions during follow‐up were handled in analyses. Treatment interruptions could affect both inflammatory marker trajectories and cardiovascular risk, potentially confounding observed associations. Future studies should incorporate repeated assessments of ART adherence and viral suppression throughout follow‐up.

Additionally, due to the small number of eligible studies per biomarker, pre‐planned subgroup and sensitivity analyses could not be performed. The included studies span periods when there was a shift towards universal test‐and‐treat strategies, as well as the adoption of integrase inhibitor treatment. Because we did not conduct a formal meta‐analysis with a subgroup analysis, we are unable to quantify this heterogeneity, yet these differences are likely to limit the comparability of findings between studies. Although ART may reduce systemic inflammation, the relationship between specific regimens, treatment duration and inflammatory biomarker levels remains complex, with evidence suggesting that biomarker trajectories vary according to regimen [5, 6, 61]. However, biomarkers may also remain elevated despite viral suppression from therapy [34, 38, 41]. Therefore, it remains challenging to interpret biomarker levels and downstream risk for individual people living with HIV and, without sensitivity analyses, we could not account for how individual and treatment‐related characteristics may have influenced findings.

The limited number of studies, particularly from low‐ and middle‐income countries (LMICs), also raises concerns about the completeness of the evidence base and the potential for publication bias. Although our review incorporated studies from multiple geographical settings, the predominance of high‐income cohorts with limited representation of women and non‐White participants raises concerns about generalisability. This demographic imbalance is particularly concerning given the disproportionate burden of CVD in PLWH in LMICs [1]. Similar systematic biases affect cardiovascular risk prediction research in this population, with a recent analysis of the REPRIEVE data showing how existing CVD risk scores underpredict risk in women and Black or African Americans living in high‐income countries [12]. Future studies should prioritise more diverse populations to ensure broader applicability of biomarker‐based risk prediction.

While our review demonstrates associations between inflammatory biomarkers and cardiovascular outcomes, translation into clinical practice will require consideration of local resources and infrastructure. Future research should not only validate these biomarkers in underrepresented populations but also assess their cost‐effectiveness and feasibility of implementation in different healthcare settings. Point‐of‐care testing technologies that can measure inflammatory markers more affordably and accessibly may ultimately be necessary to make biomarker‐based risk stratification viable in resource‐limited settings where the burden of HIV‐associated CVD is greatest.

## 5 Conclusions

As CVD continues to emerge as a leading cause of morbidity and mortality among people living with HIV, improving risk prediction remains a pressing priority. Our findings highlight that several biomarkers, including hsCRP, IL‐6, D‐dimer and NT‐proBNP, are consistently associated with an increased risk of cardiovascular disease in this population. While these associations are biologically plausible and reflect the chronic inflammation and immune activation characteristic of HIV, their predictive performance for individual risk stratification remains uncertain due to the limited number of high‐quality studies assessing each biomarker consistently across different settings and patient groups.

High‐quality, standardised longitudinal studies with consistent biomarker measurement timing and confounder adjustment are needed to better define their clinical relevance among PLWH. Future research should focus on validating promising biomarkers in more diverse populations, including women and individuals in low‐ and middle‐income settings, and integrating these markers into risk prediction models developed for PLWH. Such efforts will be critical for improving cardiovascular risk stratification and guiding targeted prevention strategies in this growing at‐risk population.

## Author Contributions

AM, RJS, TRF and JD conceptualised the design of the study. AM led the study screening and selection and the writing of the manuscript. RJS, TRF and JD served as senior reviewers on the study team. AM screened all abstracts and NL, AL, AP, NM, HP, KW and CFG contributed towards abstract screening. AM and NL performed full text screening. AM performed extraction which was checked by TRF. AM and NL performed the bias assessment. All authors contributed to the manuscript and approved the final submitted version.

## Funding

AM's PhD tuition is funded by the Oppenheimer Memorial Trust. TRF receives funding from the National Institute for Health and Care Research (NIHR) HealthTech Research Centre for Community Healthcare at Oxford Health NHS Foundation Trust (NIHR205287) and the NIHR Applied Research Collaboration Oxford and Thames Valley at Oxford Health NHS Foundation Trust (NIHR200172). JD, Academic Clinical Lecturer (CL‐2022‐13‐005), is funded by the UK NIHR. The views expressed are those of the authors and not necessarily those of the NHS, the NIHR or the Department of Health and Social Care.

## Conflicts of Interest

There are no competing interests among the authors.

## Supporting information




**Figure S1**: Forest plot of hsCRP effect estimates for major adverse cardiovascular events.

**Figure S2**: Traffic light plot of per‐study bias.

**Table S1**: Extended characteristics of included studies.

**Table S2**: Biomarkers with evidence from single studies.

**Supporting File 1**: jia270101‐sup‐0005‐SupInfo‐File‐1.docx

**Supporting File 2**: jia270101‐sup‐0006‐SupInfo‐File‐2.docx

## Data Availability

Data sharing not applicable to this article as no datasets were generated or analysed during the current study.

## References

[1] A. S. V. Shah , D. Stelzle , K. K. Lee , et al., “Global Burden of Atherosclerotic Cardiovascular Disease in People Living with the Human Immunodeficiency Virus: A Systematic Review and Meta‐Analysis,” Circulation 138, no. 11 (2018, Sept 11): 1100–1112. 29967196 10.1161/CIRCULATIONAHA.117.033369 PMC6221183

[2] L. Mavarani , N. Reinsch , S. Albayrak‐Rena , et al., “The Association of HIV‐Specific Risk Factors with Cardiovascular Events in Addition to Traditional Risk Factors in People Living with HIV,” AIDS Research and Human Retroviruses 40, no. 4 (2024 Apr): 235–245. 37675901 10.1089/AID.2023.0055

[3] S. Dirajlal‐Fargo and N. Funderburg , “HIV and Cardiovascular Disease: The Role of Inflammation,” Current Opinion in HIV and AIDS 17, no. 5 (2022 Sep): 286. 35938462 10.1097/COH.0000000000000755 PMC9370832

[4] L. M. Obare , T. Temu , S. A. Mallal , and C. N. Wanjalla , “Inflammation in HIV and Its Impact on Atherosclerotic Cardiovascular Disease,” Circulation Research 134, no. 11 (2024 May 24): 1515–1545. 38781301 10.1161/CIRCRESAHA.124.323891 PMC11122788

[5] A. Kearns , J. Gordon , T. H. Burdo , and X. Qin , “HIV‐1–Associated Atherosclerosis: Unraveling the Missing Link,” Journal of the American College of Cardiology 69, no. 25 (2017 June 27): 3084–3098. 28641798 10.1016/j.jacc.2017.05.012 PMC5512584

[6] S. Zicari , L. Sessa , N. Cotugno , et al., “Immune Activation, Inflammation, and Non‐AIDS Co‐Morbidities in HIV‐Infected Patients under Long‐Term ART,” Viruses 11, no. 3 (2019 Feb 27): 200. 30818749 10.3390/v11030200 PMC6466530

[7] C. C. Esenwa and M. S. Elkind , “Inflammatory Risk Factors, Biomarkers and Associated Therapy in Ischaemic Stroke,” Nature Reviews Neurology 12, no. 10 (2016 Oct): 594–604. 27615422 10.1038/nrneurol.2016.125

[8] S. Kinlay and J. Egido , “Inflammatory Biomarkers in Stable Atherosclerosis,” The American Journal of Cardiology 98, no. 11 (2006 Dec 4): S2–8. 10.1016/j.amjcard.2006.09.014 17126676

[9] X. H. Chen , S. Huang , and D. Kerr , “Biomarkers in Clinical Medicine,” IARC Scientific Publications no. 163 (2011): 303–322. 22997869

[10] A. C. Achhra , A. Lyass , L. Borowsky , et al., “Assessing Cardiovascular Risk in People Living with HIV: Current Tools and Limitations,” Current HIV/AIDS Reports 18, no. 4 (2021 Aug): 271–279. 34247329 10.1007/s11904-021-00567-w PMC8733948

[11] C. Soares , M. Kwok , K. A. Boucher , et al., “Performance of Cardiovascular Risk Prediction Models Among People Living With HIV: A Systematic Review and Meta‐analysis,” JAMA Cardiology 8, no. 2 (2023 Feb 1): 139–149. 36576812 10.1001/jamacardio.2022.4873 PMC9857084

[12] S. K. Grinspoon , M. V. Zanni , V. A. Triant , et al., “Performance of the Pooled Cohort Equations and D:A:D Risk Scores among Individuals with HIV in a Global Cardiovascular Disease Prevention Trial: A Cohort Study Leveraging Data from REPRIEVE,” The Lancet HIV 12, no. 2 (2025 Feb 1): e118–e129. 39832519 10.1016/S2352-3018(24)00276-5 PMC11890582

[13] M. Durand , “Risk Estimation in HIV Reveals Our Usual Blind Spots,” The Lancet HIV 12, no. 2 (2025 Jan 17): e85–e86. 39832520 10.1016/S2352-3018(24)00351-5

[14] R. D. Riley , K. G. M. Moons , K. I. E. Snell , et al., “A Guide to Systematic Review and Meta‐analysis of Prognostic Factor Studies,” BMJ 364 (2019 Jan 30): k4597. 30700442 10.1136/bmj.k4597

[15] D. J. Medina‐Leyte , O. Zepeda‐García , M. Domínguez‐Pérez , A. González‐Garrido , T. Villarreal‐Molina , and L. Jacobo‐Albavera , “Endothelial Dysfunction, Inflammation and Coronary Artery Disease: Potential Biomarkers and Promising Therapeutical Approaches,” International Journal of Molecular Sciences 22, no. 8 (2021 Apr 8): 3850. 33917744 10.3390/ijms22083850 PMC8068178

[16] D. R. Germolec , K. A. Shipkowski , R. P. Frawley , and E. Evans , “Markers of Inflammation,” Methods in Molecular Biology 1803 (2018): 57–79. 29882133 10.1007/978-1-4939-8549-4_5

[17] R. S. Vasan , “Biomarkers of Cardiovascular Disease,” Circulation 113, no. 19 (2006 May 16): 2335–2362. 16702488 10.1161/CIRCULATIONAHA.104.482570

[18] S. R. Das , B. M. Everett , K. K. Birtcher , et al., “2018 ACC Expert Consensus Decision Pathway on Novel Therapies for Cardiovascular Risk Reduction in Patients With Type 2 Diabetes and Atherosclerotic Cardiovascular Disease: A Report of the American College of Cardiology Task Force on Expert Consensus Decision Pathways,” Journal of the American College of Cardiology 72, no. 24 (2018): 3200–3223. 30497881 10.1016/j.jacc.2018.09.020 PMC7560953

[19] J. Remick , V. Georgiopoulou , C. Marti , et al., “Heart Failure in Patients With Human Immunodeficiency Virus Infection: Epidemiology, Pathophysiology, Treatment, and Future Research,” Circulation 129, no. 17 (2014 Apr 29): 1781–1789. 24778120 10.1161/CIRCULATIONAHA.113.004574 PMC4006343

[20] J. A. Hayden , D. A. van der Windt , J. L. Cartwright , P. Côté , and C. Bombardier , “Assessing Bias in Studies of Prognostic Factors,” Annals of Internal Medicine 158, no. 4 (2013 Feb 19): 280–286. 23420236 10.7326/0003-4819-158-4-201302190-00009

[21] A. Iorio , F. A. Spencer , M. Falavigna , et al., “Use of GRADE for Assessment of Evidence about Prognosis: Rating Confidence in Estimates of Event Rates in Broad Categories of Patients,” BMJ 350 (2015 Mar 16): h870. 25775931 10.1136/bmj.h870

[22] S. A. R. Doi , J. J. Barendregt , S. Khan , L. Thalib , and G. M. Williams , “Advances in the Meta‐analysis of Heterogeneous Clinical Trials I: The Inverse Variance Heterogeneity Model,” Contemporary Clinical Trials 45 (2015 Nov 1): 130–138. 26003435 10.1016/j.cct.2015.05.009

[23] J. P. T. Higgins , J. Thomas , J. Chandler , et al., eds, Cochrane Handbook for Systematic Reviews of Interventions (John Wiley & Sons, 2019).

[24] R Core Team , R: A Language and Environment for Statistical Computing (R Foundation for Statistical Computing, 2021), https://www.R‐project.org/.

[25] D. Fisher , ADMETAN: Stata Module to Provide Comprehensive Meta‐Analysis, Boston College Department of Economics (Statistical Software Components S458561, 2018).

[26] StataCorp , Stata Statistical Software: Release 18, Texas College Station (StataCorp LLC, 2023).

[27] L. A. McGuinness and J. P. T. Higgins , “Risk‐of‐Bias VISualization (robvis): An R Package and Shiny Web App for Visualizing Risk‐of‐Bias Assessments,” Research Synthesis Methods 12 (2020): 55–61. 32336025 10.1002/jrsm.1411

[28] M. J. Page , J. E. McKenzie , P. M. Bossuyt , et al., “The PRISMA 2020 Statement: An Updated Guideline for Reporting Systematic Reviews,” BMJ 372 (2021 Mar 29): n71. 33782057 10.1136/bmj.n71 PMC8005924

[29] A. Knudsen , T. L. Katzenstein , T. Benfield , et al., “Plasma Plasminogen Activator Inhibitor‐1 Predicts Myocardial Infarction in HIV‐1‐infected Individuals,” AIDS 28, no. 8 (2014 May 15): 1171–1179. 24566095 10.1097/QAD.0000000000000247

[30] A. Knudsen , H. J. Møller , T. L. Katzenstein , et al., “Soluble CD163 Does Not Predict First‐time Myocardial Infarction in Patients Infected with human Immunodeficiency Virus: A Nested Case–control Study,” BMC Infectious Diseases 13, no. 1 (2013 May 21): 230. 23692821 10.1186/1471-2334-13-230 PMC3663777

[31] J. M. Haissman , A. Knudsen , H. Hoel , et al., “Microbiota‐Dependent Marker TMAO Is Elevated in Silent Ischemia but Is Not Associated With First‐Time Myocardial Infarction in HIV Infection,” Journal of Acquired Immune Deficiency Syndromes 71, no. 2 (2016 Feb 1): 130. 26413854 10.1097/QAI.0000000000000843

[32] H. Hoel , T. Ueland , A. Knudsen , et al., “Soluble Markers of Interleukin 1 Activation as Predictors of First‐Time Myocardial Infarction in HIV‐Infected Individuals,” The Journal of Infectious Diseases 221, no. 4 (2020 Feb 3): 506–509. 31077280 10.1093/infdis/jiz253

[33] L. Rasmussen , A. Knudsen , T. Katzenstein , et al., “Soluble Urokinase Plasminogen Activator Receptor (suPAR) Is a Novel, Independent Predictive Marker of Myocardial Infarction in HIV‐1‐infected Patients: A Nested Case‐control Study,” HIV Medicine 17, no. 5 (2016): 350–357. 26365671 10.1111/hiv.12315 PMC5054925

[34] A. R. Tenorio , Y. Zheng , R. J. Bosch , et al., “Soluble Markers of Inflammation and Coagulation but Not T‐Cell Activation Predict Non–AIDS‐Defining Morbid Events During Suppressive Antiretroviral Treatment,” The Journal of Infectious Diseases 210, no. 8 (2014 Oct 15): 1248–1259. 24795473 10.1093/infdis/jiu254 PMC4192039

[35] T. A. Premeaux , C. B. Moser , A. McKhann , et al., “Plasma Galectin‐9 as a Predictor of Adverse Non‐AIDS Events in Persons with Chronic HIV during Suppressive Antiretroviral Therapy,” AIDS 35, no. 15 (2021 Dec 1): 2489. 34366381 10.1097/QAD.0000000000003048 PMC8631144

[36] ÁH Borges , J. L. O'Connor , and A. N. Phillips , “Interleukin 6 Is a Stronger Predictor of Clinical Events Than High‐Sensitivity C‐Reactive Protein or D‐Dimer During HIV Infection,” Journal of Infectious Diseases 214, no. 3 (2016 Aug 1): 408–416. 27132283 10.1093/infdis/jiw173 PMC4936649

[37] D. A. Duprez , J. Neuhaus , R. Tracy , et al., “N‐terminal‐proB‐type Natriuretic Peptide Predicts Cardiovascular Disease Events in HIV‐infected Patients,” AIDS 25, no. 5 (2011 Mar 13): 651. 21245726 10.1097/QAD.0b013e32834404a1 PMC3113476

[38] D. A. Duprez , J. Neuhaus , L. H. Kuller , et al., “Inflammation, Coagulation and Cardiovascular Disease in HIV‐infected Individuals,” PLoS ONE 7, no. 9 (2012): e44454. 22970224 10.1371/journal.pone.0044454 PMC3438173

[39] D. Duprez , J. Neuhaus , J. Otvos , J. D. Neaton , and J. D. Lundgren , “Abstract 14731: Glyc A, a Novel Marker of Inflammation, Predicts Cardiovascular Events in HIV‐Positive Patients: Results of SMART Study,” Circulation 130, no. suppl_2 (2014 Nov 25): A14731–A14731.

[40] S. E. Safo , L. Haine , J. Baker , et al., “Derivation of a Protein Risk Score for Cardiovascular Disease Among a Multiracial and Multiethnic HIV+ Cohort,” Journal of the American Heart Association 12, no. 13 (2023 July 4): e027273. 37345752 10.1161/JAHA.122.027273 PMC10356060

[41] B. Grund , J. V. Baker , S. G. Deeks , et al., “Relevance of Interleukin‐6 and D‐Dimer for Serious Non‐AIDS Morbidity and Death among HIV‐Positive Adults on Suppressive Antiretroviral Therapy,” PLoS ONE 11, no. 5 (2016 May 12): e0155100. 27171281 10.1371/journal.pone.0155100 PMC4865234

[42] J. I. Bernardino , B. Alejos , and J. Rodriguez‐Centeno , “Monocyte Activation and Ageing Biomarkers in the Development of Cardiovascular Ischaemic Events or Diabetes in People with HIV,” Microorganisms 11, no. 7 (2023 July 16): 1818. 37512990 10.3390/microorganisms11071818 PMC10385988

[43] P. de Leuw , C. T. Arendt , A. E. Haberl , et al., “Myocardial Fibrosis and Inflammation by CMR Predict Cardiovascular Outcome in People Living With HIV,” JACC: Cardiovascular Imaging 14, no. 8 (2021 Aug 1): 1548–1557. 33865770 10.1016/j.jcmg.2021.01.042

[44] V. C. Marconi , M. S. Duncan , K. So‐Armah , et al., “Bilirubin Is Inversely Associated With Cardiovascular Disease Among HIV‐Positive and HIV‐Negative Individuals in VACS (Veterans Aging Cohort Study),” Journal of the American Heart Association 7, no. 10 (2018 May 15): e007792. 29720501 10.1161/JAHA.117.007792 PMC6015337

[45] A. O. Mocumbi , I. Dobe , S. Cândido , and N. Kim , “Cardiovascular Risk and D‐dimer Levels in HIV‐infected ART‐naïve Africans,” Cardiovascular Diagnosis and Therapy 10, no. 3 (2020): 526–533. 32695632 10.21037/cdt.2019.12.02 PMC7369281

[46] E. S. Ford , J. H. Greenwald , A. G. Richterman , et al., “Traditional Risk Factors and D‐dimer Predict Incident Cardiovascular Disease Events in Chronic HIV Infection,” AIDS 24, no. 10 (2010 June 19): 1509–1517. 20505494 10.1097/QAD.0b013e32833ad914 PMC2884071

[47] C. Missailidis , U. Neogi , P. Stenvinkel , M. Trøseid , P. Nowak , and P. Bergman , “The Microbial Metabolite Trimethylamine‐N‐oxide in Association with Inflammation and Microbial Dysregulation in Three HIV Cohorts at Various Disease Stages,” AIDS 32, no. 12 (2018 July 31): 1589. 29620717 10.1097/QAD.0000000000001813

[48] N. Reinsch , H. Streeck , V. Holzendorf , et al., “B‐type Natriuretic Peptides for the Prediction of Cardiovascular Events and Mortality in Patients Living with HIV: Results from the HIV‐HEART Study,” International Journal of Cardiology 281 (2019 Apr 15): 127–132. 30711264 10.1016/j.ijcard.2019.01.066

[49] D. M. Kirkegaard‐Klitbo , N. Mejer , T. B. Knudsen , et al., “Soluble CD163 Predicts Incident Chronic Lung, Kidney and Liver Disease in HIV Infection,” AIDS 31, no. 7 (2017 Apr 24): 981. 28252527 10.1097/QAD.0000000000001432

[50] A. G. Vos , N. S. Idris , R. E. Barth , K. Klipstein‐Grobusch , and D. E. Grobbee , “Pro‐Inflammatory Markers in Relation to Cardiovascular Disease in HIV Infection. A Systematic Review,” PLoS ONE 11, no. 1 (2016 Jan 25): e0147484. 26808540 10.1371/journal.pone.0147484 PMC4726827

[51] J. Hartman and W. H. Frishman , “Inflammation and Atherosclerosis: A Review of the Role of Interleukin‐6 in the Development of Atherosclerosis and the Potential for Targeted Drug Therapy,” Cardiology in Review 22, no. 3 (2014 June): 147. 24618929 10.1097/CRD.0000000000000021

[52] S. G. Deeks , R. Tracy , and D. C. Douek , “Systemic Effects of Inflammation on Health during Chronic HIV Infection,” Immunity 39, no. 4 (2013 Oct 17): 633–645. 24138880 10.1016/j.immuni.2013.10.001 PMC4012895

[53] T. Banait , A. Wanjari , V. Danade , S. Banait , and J. Jain , “Role of High‐Sensitivity C‐reactive Protein (hs‐CRP) in Non‐communicable Diseases: A Review,” Cureus 14, no. 10: e30225. 36381804 10.7759/cureus.30225 PMC9650935

[54] H. M. Spronk , D. van der Voort , and H. Ten Cate , “Blood Coagulation and the Risk of Atherothrombosis: A Complex Relationship,” Thrombosis Journal 2, no. 1 (2004 Dec 1): 12. 15574198 10.1186/1477-9560-2-12 PMC538274

[55] H. Jouni , R. J. Rodeheffer , and I. J. Kullo , “Increased Serum N‐Terminal Pro–B‐Type Natriuretic Peptide Levels in Patients With Medial Arterial Calcification and Poorly Compressible Leg Arteries,” Arteriosclerosis, Thrombosis, and Vascular Biology 31, no. 1 (2011 Jan): 197–202. 20947817 10.1161/ATVBAHA.110.216770 PMC3115928

[56] J. B. Tcheugui , S. Zhang , J. W. McEvoy , et al., “Elevated NT‐ProBNP as a Cardiovascular Disease Risk Equivalent: Evidence from the Atherosclerosis Risk in Communities (ARIC) Study,” American Journal of Medicine 135, no. 12 (2022 Dec): 1461–1467. 36007589 10.1016/j.amjmed.2022.07.012 PMC10208080

[57] Y. Liu , S. Guan , H. Xu , N. Zhang , M. Huang , and Z. Liu , “Inflammation Biomarkers Are Associated with the Incidence of Cardiovascular Disease: A Meta‐analysis,” Frontiers in Cardiovascular Medicine 10 (2023): 1175174. 37485268 10.3389/fcvm.2023.1175174 PMC10360053

[58] T. Shah , J. P. Casas , J. A. Cooper , et al., “Critical Appraisal of CRP Measurement for the Prediction of Coronary Heart Disease Events: New Data and Systematic Review of 31 Prospective Cohorts,” International Journal of Epidemiology 38, no. 1 (2009 Feb 1): 217–231. 18930961 10.1093/ije/dyn217 PMC2639366

[59] A. A. Ashuro , Y. G. Fan , Y. S. Fu , et al., “The Effect of Rosuvastatin on Plasma/Serum Levels of High‐Sensitivity C‐Reactive Protein, Interleukin‐6, and D‐Dimer in People Living with Human Immunodeficiency Virus: A Systematic Review and Meta‐Analysis,” AIDS Research and Human Retroviruses 37, no. 11 (2021 Nov): 821–833. 33913752 10.1089/AID.2020.0273

[60] N. Rifai , D. G. Altman , and P. M. Bossuyt , “Reporting Bias in Diagnostic and Prognostic Studies: Time for Action,” Clinical Chemistry 54, no. 7 (2008 July 1): 1101–1103. 18593957 10.1373/clinchem.2008.108993

[61] R. M. Alvi , M. V. Zanni , A. M. Neilan , et al., “Amino‐terminal Pro‐B‐Type Natriuretic Peptide Among Patients Living With both Human Immunodeficiency Virus and Heart Failure,” Clinical Infectious Diseases 71, no. 5 (2020 Aug 22): 1306–1315. 31740919 10.1093/cid/ciz958 PMC7442853

[62] O. G. Goryacheva and N. A. Koziolova , “Risk Factors of Severe Heart Failure in HIV‐positive Patients,” Russian Journal of Cardiology 26, no. 1 (2021 Jan 6): 4275.

