# Supplementary material for: Inflammatory Biomarkers Predicting Major Adverse Cardiovascular Events in People Living With HIV: A Systematic Review and Meta‐Analysis
Source: J Int AIDS Soc. 2026 Apr 27;29(4):e70101. doi: 10.1002/jia2.70101 (PMC13113420; doi:10.1002/jia2.70101)
Supplement: Supplementary file 1 — Figure S1: Forest plot of hsCRP effect estimates for major adverse cardiovascular events. [file JIA2-29-e70101-s004.docx]

**Supporting Figure S1. “Inflammatory biomarkers predicting cardiovascular events in people living with HIV: a systematic review and meta-analysis”**

**
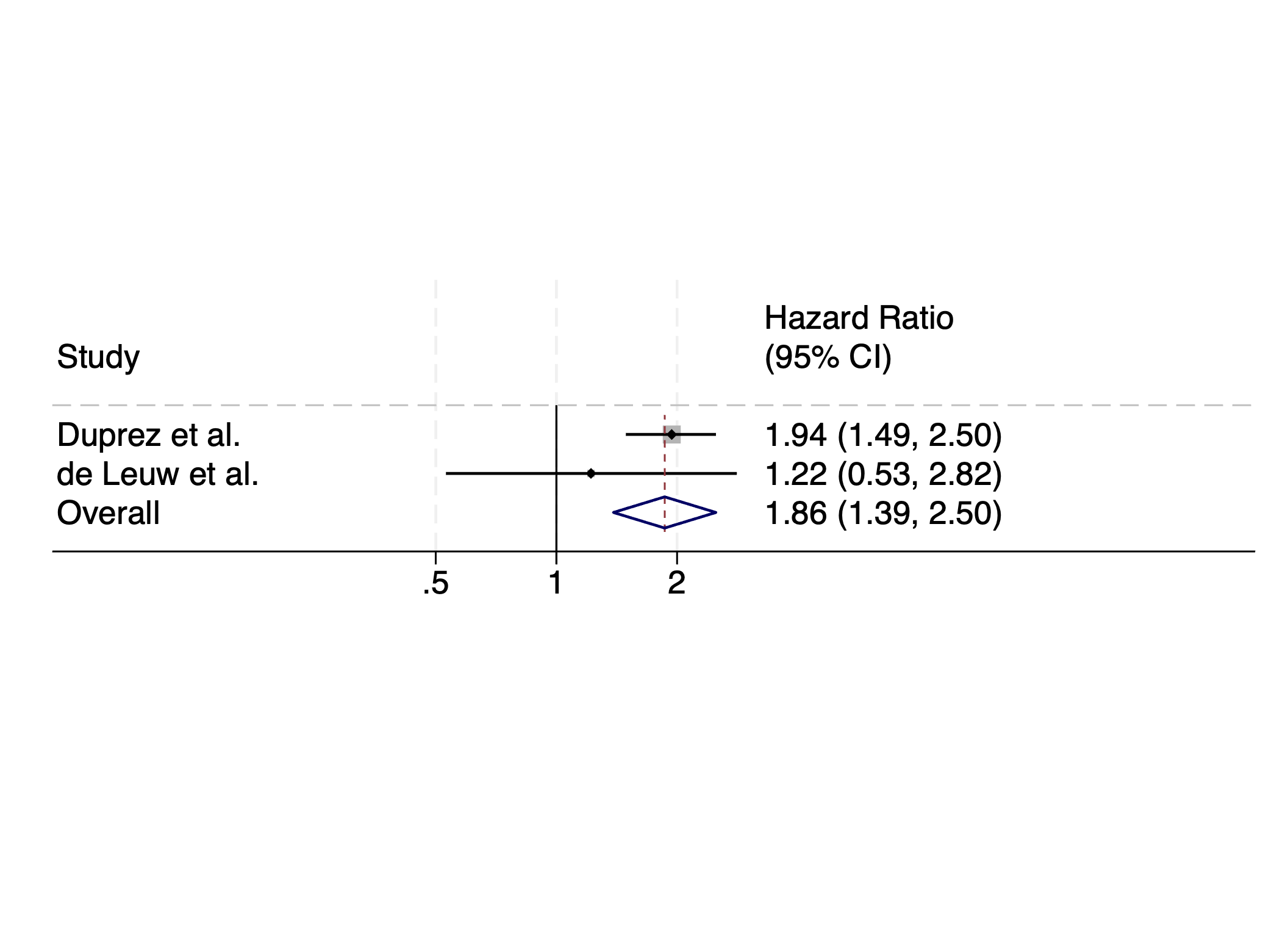
**

**Supporting Figure S1. Forest plot of hsCRP effect estimates for major adverse cardiovascular events.**
