# Supplementary material for: Inflammatory Biomarkers Predicting Major Adverse Cardiovascular Events in People Living With HIV: A Systematic Review and Meta‐Analysis
Source: J Int AIDS Soc. 2026 Apr 27;29(4):e70101. doi: 10.1002/jia2.70101 (PMC13113420; doi:10.1002/jia2.70101)
Supplement: Supplementary file 2 — Figure S2: Traffic light plot of per‐study bias. [file JIA2-29-e70101-s003.docx]

**Supporting Figure S2. “Inflammatory biomarkers predicting cardiovascular events in people living with HIV: a systematic review and meta-analysis”**


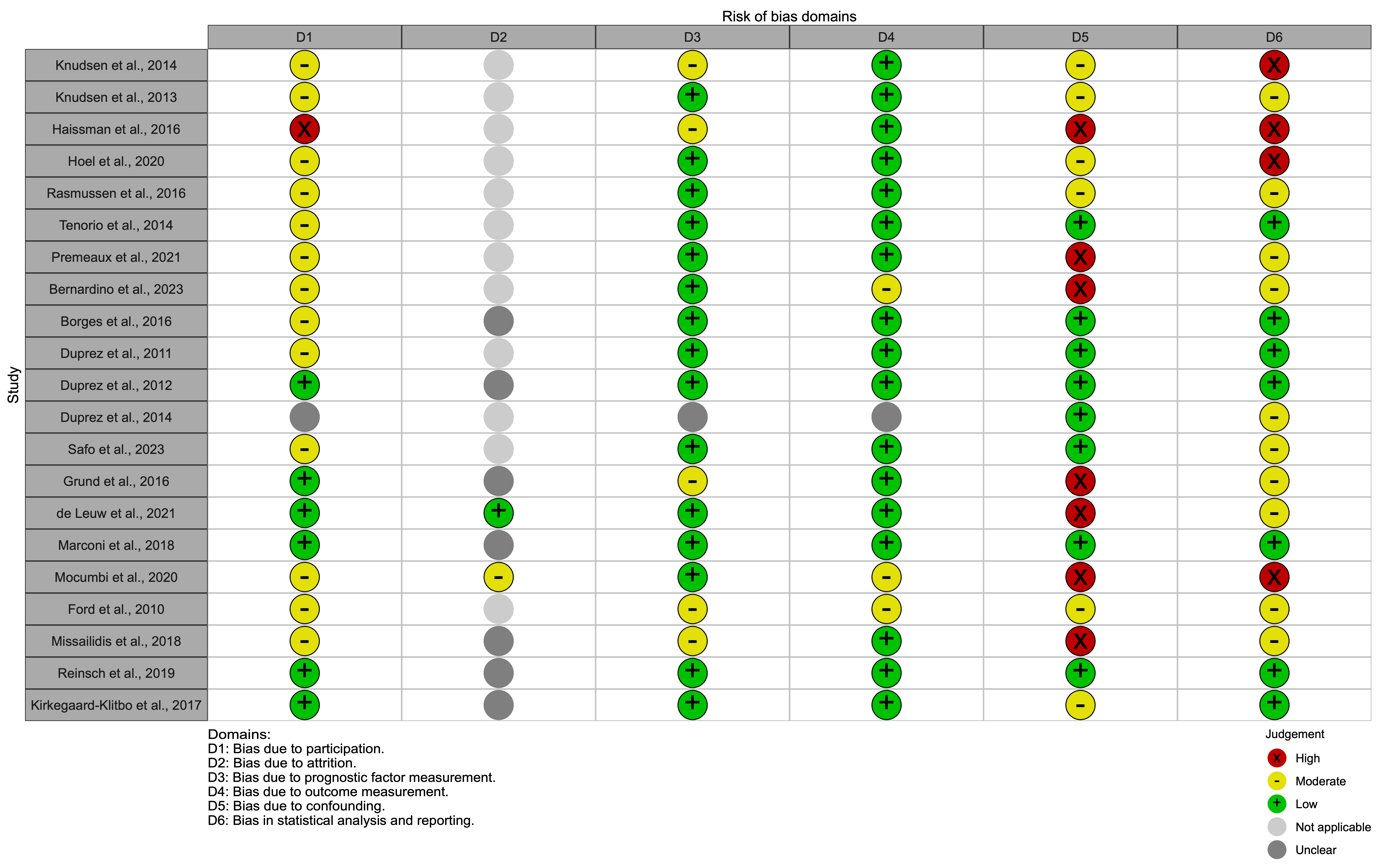


**Supporting Figure S2. Traffic light plot of per-study bias**
