# Supplementary material for: Inflammatory Biomarkers Predicting Major Adverse Cardiovascular Events in People Living With HIV: A Systematic Review and Meta‐Analysis
Source: J Int AIDS Soc. 2026 Apr 27;29(4):e70101. doi: 10.1002/jia2.70101 (PMC13113420; doi:10.1002/jia2.70101)
Supplement: Supplementary file 3 — Table S1: Extended characteristics of included studies. [file JIA2-29-e70101-s001.docx]

**Supporting Table S1: “Inflammatory biomarkers predicting cardiovascular events in people living with HIV: a systematic review and meta-analysis”**

**Table S1. Extended characteristics of included studies**

| **Authors (Year)** | **Location(s)** | **Study design** | **Sample size (n)^†^** | **Female (n, %)** | **Ethnicity (n, %)** | **Eligible biomarker(s) reported** | **Number of events (n)^†^** | **Outcome definition** | **VL at biomarker measurement (copies/ml)^†‡^** | **VL proximal to event (copies/ml)** **^‡^** | **Follow-up** | **Biomarker collection timepoints** |
| --- | --- | --- | --- | --- | --- | --- | --- | --- | --- | --- | --- | --- |
| **Danish HIV Cohort Study** |  |  |  |  |  |  |  |  |  |  |  |  |
| Knudsen et al. (2014) | Denmark | CC | Cases: 54 Controls: 54 Total: 108 | 8, 7.4 | NR | hsCRP, PAI-1 | 54 | MI | NR | Cases: 39, 19-217000 Controls: 31, 19-29700  [2 months pre-event] | NA | 12 & 2 months pre-event |
| Knudsen et al. (2013) | Denmark | CC | Cases: 55 Controls: 182 Total: 237 | 20, 8.4 | Caucasian: 222, 93.7 | sCD163 | 55 | MI | NR | Cases: 39, 19–217200 Controls: 39, 19–93900  [52 days pre-event] | NA | Pre-ART, 3 months post-ART, 1 year & 52 days pre-event |
| Haissman et al. (2016) | Denmark | CC | Cases: 49  Controls: 160  Total: 209 | 18, 8.3 | NR | TMAO | 49 | MI | NR (only pre-ART) | NR | NA | Pre-ART, 3 months post-ART, 1 year & 51 days pre-event |
| Hoel et al. (2020) | Denmark | CC | Cases: 55  Controls: 182  Total: 237 | 20, 8.4 | NR | IL-1Ra, IL-1R1 | 55 | MI | Cases: 199, 28-399  Controls: 199, 20-399 | Cases: 39, 19-92  Controls: 39, 19-40  [52 days pre-event] | NA | Pre-ART, 3 months post-ART, 1 year & 52 days pre-event |
| Rasmussen et al. (2016) | Denmark | CC | Cases: 55 Controls: 182 Total: 237 | 20, 8.4 | NR | suPAR | 55 | MI | Cases: 199, 28-399  Controls: 199, 20-399 | Cases: 39, 19-92  Controls: 39, 19-40  [52 days pre-event] | NA | Pre-ART, 3 months post-ART, 1 year & 52 days pre-event |
| **AIDS Clinical Trials Group Longitudinal Linked Randomized Trials** |  |  |  |  |  |  |  |  |  |  |  |  |
| Tenorio et al. (2014) | USA | CC | Cases: 41  Controls: 94  Total: 135 | 70, 15 | White: 225, 49 Black: 141, 31 Hispanic: 80, 17 Other: 12, 3 | D-dimer, IL-6, IP-10, sTNFR-I, sTNFR-II, sCD14 | 41 | MI, CVA | NR (only pre-ART) | NR | NA | Pre-ART, 48-64 weeks post-ART, pre-event |
| Premeaux et al. (2021) | USA | CC | Cases: 32  Controls: 62  Total: 94 | 67, 16 | White: 208, 49 Black: 130, 31 Hispanic: 76, 18 Other: 12, 2.8 | Gal-9 | 32 | MI, CVA | NR (only pre-ART) | NR | NA | Pre-ART, 48-64 weeks post-ART & 10.5 weeks pre-event |
| **Strategies for Management of Antiretroviral Therapy Study** |  |  |  |  |  |  |  |  |  |  |  |  |
| Borges et al. (2016) | Global | COH | SMART: 2526  Total: 4304 | 1002, 23.3 | Black: 907, 21.1 | D-dimer, hsCRP, IL-6 | Overall: 121 SMART: 62 | MI, CVA, or CHD requiring a surgical procedure | ≤ 500 copies/ml: n = 3263 (75.8%); no median or mean reported | NR | Median 4 years | Baseline |
| Duprez et al. (2011) | Global | CC | Cases: 186 Controls: 329 Total: 515 | 102, 19.8 | Black: 197, 38.2 | NT-proBNP | 186 | CHD, CVA, PAD, CHF, CVD death | ≤ 400 copies/ml: n = 360 (69.9%); no median or mean reported | NR | Mean 2.8 years | Baseline |
| Duprez et al. (2012) | Global | COH | 5098 | 1322, 25.9 | Black: 1491, 29.24 | D-dimer, hsCRP, IL-6 | 252 | CHD, MI, CVA, coronary revascularisation, PAD, CHF, CVD death | ≤ 400 copies/ml: n = 3621 (71%); no median or mean reported | NR | Median 2.4 yrs | Baseline |
| Duprez et al. (2014) | Global | CC | Cases: 246 Controls: 472 Total: 718 | NR | NR | Glyc A | 246 | CHD, MI, CVA, coronary revascularisation, PAD, CHF, CVD death | NR | NR | “Average” 2.8 years | Baseline |
| Safo et al. (2023) | Global | CC | ESPRIT: 228 FIRST: 24 SMART: 48 START: 90  Total: 390 | 49, 12.6 | Black: 66, 16.9 | IL-6, ITGA11,  CCL25, PLA2G7 | ESPRIT: 77  FIRST: 8  SMART: 16  START: 30  Overall: 131 | MI, CVA, coronary revascularisation, CAD requiring surgery, CVD death | Cases: 35856.41 ± 224730.34  Controls: 15779.12 ± 57288.42 | NR | NA | Baseline |
| Grund et al. (2016) | Global | COH | SMART: 1748 ESPRIT: 1446 SILCAAT: 572  Total: 3766 | 799, 21.2 | Black: 618, 16.4 | D-dimer, hsCRP, IL-6 | 84 | MI, CVA, CVD death | ≤ 500 copies/ml: n = 3766 (100%); no median or mean reported | NR | Mean 4.9 years | Baseline |
| Bernardino et al. (2023)^§^ | Spain | CC | Cases: 34  Controls: 34  Total: 68 | 30, 16 | Caucasian: 156, 83 | sCD14, sCD163, CCL2 | 34 | MI, CVA, and ‘sudden’ deaths ascribed by authors as being ischaemic events | Cases: 33884.4, 4466.8–117489.7 Controls: 30902.9, 7079.5–134896.3 | NR | NA | 2.4 years pre-event |
| de Leuw et al. (2021) | Germany | COH | 156 | 59, 37.8 | White: 122, 78.2 | hsCRP, hsTNT, NT-proBNP | 24 | ACS or an appropriate device discharge, HF hospitalisation, CVD death | NR | NR | Median 13 months | Baseline |
| Marconi et al. (2018)^§^ | USA | COH | 30427 | 2891, 3 | Black: 46262, 48 | Total bilirubin | 2355 | MI, CVA, HF | Quartile 1 of bilirubin (≤0.4 mg/dL): 73709.8 ± 246747.5  Quartile 2 of bilirubin (0.5–0.6 mg/dL): 91714.1 ± 1800754.0  Quartile 3 of bilirubin (0.7–0.8 mg/dL): 58102.7 ± 224687.8  Quartile 4 of bilirubin (≥0.9 mg/dL): 45276.0 ± 195459.9  Missing bilirubin: 59227.0 ± 162158.5 | NR | Mean 5.7 years | Baseline |
| Mocumbi et al. (2020) | Mozambique | COH | 70 | 41, 58.6 | Black: 70, 100 | D-dimer | 4 | CVA, HF | NR | NR | 3 years | Baseline |
| Ford et al. (2010) | USA | CC | Cases: 52 Controls: 104 Total: 156 | 3, 1.9 | African American: 25, 16.0 | D-dimer, hsCRP, IL-6, VCAM-1, ICAM-1, TIMP-1, MPO, IL-2, IL-10, sCD14, TNF-α, TNF-γ | 52 | MI, ACS, coronary revascularisation, CVA, PAD, CVD death | NR (only peak viral load) | Cases: 2540 ± 1570  Controls: 13860 ± 3790  [4 months pre-event] | NA | 4 months & 2 years pre-event |
| Missailidis et al. (2018)^¶^ | Sweden | COH | 101 | 4, 4 | Caucasian: 100, 100 | TMAO | 6 | Unstable angina, MI, CVA, TIA | <20 copies/ml: n = 101 (100%) | NR | 6 years | Baseline |
| Reinsch et al. (2019) | Germany | COH | 808 | 136, 16.8 | Caucasian: 719, 89 | BNP | 158 | MI, CHD, coronary artery bypass grafting, revascularisation, CVA, TIA, and PAD | <50 copies/ml: n = 423 (53.7%) | NR | Median 10 years | Baseline |
| Kirkegaard-Klitbo et al. (2017) | Denmark | COH | 799 | 210, 26.2 | Caucasian: 633, 79 Black: 102, 13 Asian: 445, 6 Other: 19, 2 | sCD163 | 84 reported from multivariate analysis  94 reported from univariate analysis | IHD, cerebrovascular disease | 19, 19-20 | NR | Median 10.5 years | Baseline |

^†^Details of post-antiretroviral cohort for case-control studies shown, where applicable. For cohort studies, baseline enrolment details are reported.

^‡^Viral load values reported by original studies as median (interquartile range) or mean ± standard deviation, unless otherwise stated

^§^Participant characteristics are from larger cohort in which cohort of interest was nested, as characteristics of cohort of interest were not available for extraction

^¶^ Only the chronic ART cohort was included as they had long-term, longitudinal follow-up

Abbreviations: ACS, Acute Coronary Syndrome; ART, Antiretroviral Therapy; BNP, B-type Natriuretic Peptide; CAD, Coronary Artery Disease; CC, Case-Control; CCL2, Chemokine Ligand 2; CCL25, Chemokine Ligand 25; CHF, Congestive Heart Failure; CHD, Coronary Heart Disease; COH, Cohort Study; CRP/hsCRP, (High-Sensitivity) C-Reactive Protein; CVA, Cerebrovascular Accident; CVD, Cardiovascular Disease; Gal-9, Galectin-9; GM-CSF, Granulocyte-Macrophage Colony-Stimulating Factor; HF, Heart Failure; ICAM-1, Intercellular Adhesion Molecule 1; IFN-γ, Interferon Gamma; IL, IHD, Ischaemic Heart Disease; Interleukin; IL-1Ra, IL-1 Receptor Antagonist; IL-1R1, IL-1 Receptor 1; IP-10, Interferon Gamma-Induced Protein 10; ITGA11, Integrin Subunit Alpha 11; MI, Myocardial Infarction; MPO, Myeloperoxidase; NA, Not Applicable; NR, Not Reported; NT-proBNP, N-terminal Pro B-type Natriuretic Peptide; PAD, Peripheral Arterial Disease; PAI-1, Plasminogen Activator Inhibitor-1; PLA2G7, Phospholipase A2 Group VII.; sCD, Soluble Cluster of Differentiation; sTNFR-I/sTNFR-II, Soluble TNF Receptors I and II; suPAR, Soluble Urokinase-Type Plasminogen Activator Receptor; TIA, Transient Ischaemic Attack; TIMP-1, Tissue Inhibitor of Metalloproteinases-1; TNT/hsTNT, (High Sensitivity) Troponin T; TNF-α, Tumor Necrosis Factor Alpha; TMAO, Trimethylamine N-oxide; VCAM-1, Vascular Cell Adhesion Molecule 1; VL, Viral Load.
