# Supplementary material for: Inflammatory Biomarkers Predicting Major Adverse Cardiovascular Events in People Living With HIV: A Systematic Review and Meta‐Analysis
Source: J Int AIDS Soc. 2026 Apr 27;29(4):e70101. doi: 10.1002/jia2.70101 (PMC13113420; doi:10.1002/jia2.70101)
Supplement: Supplementary file 4 — Table S2: Biomarkers with evidence from single studies. [file JIA2-29-e70101-s002.docx]

**Supporting Table S2: “Inflammatory biomarkers predicting cardiovascular events in people living with HIV: a systematic review and meta-analysis”**

**Table S2. Biomarkers with evidence from single studies**

| **Biomarker** | **Study** | **Association (95% CI)** | **Comparison** |
| --- | --- | --- | --- |
| PAI-1 | Knudsen et al., 2014 | aOR = 1.01 (1.00–1.03) | Per ng/ml |
| IL-1Ra | Hoel et al., 2020^†^ | aOR = 1.63 (1.13–2.36) | Per pg/mL |
| IL-1R1 | Hoel et al., 2020^†^ | aOR = 1.22 (0.83–1.78) | Per pg/mL |
| suPAR | Rasmussen et al., 2016^†^ | aOR = 1.27 (1.01–1.61) | Per ng/mL |
| IP-10 | Tenorio et al., 2014^†^ | aOR = 2.6 (0.6–11.1) | Per log_10_ |
| sTNFR-I | Tenorio et al., 2014^†^ | aOR = 218 (4.6–1.0×10⁴) | Per log_10_ |
| sTNFR-II | Tenorio et al., 2014^†^ | aOR = 50.4 (2.9–886) | Per log_10_ |
| Gal-9 | Premeaux et al., 2021^†^ | OR = 22.2 (1.4–356.8) | Per log_10_ |
| Glyc A | Duprez et al., 2014 | aOR = 2.20 (1.29–3.76) | Highest vs lowest quartile^‡^ |
| ITGA11 | Safo et al., 2023 | aOR = 0.85 (0.66-1.09) | Per 1 SD in NPX |
| CCL25 | Safo et al., 2023 | aOR = 1.26 (0.99-1.61) | Per 1 SD in NPX |
| PLA2G7 | Safo et al., 2023 | aOR = 1.48 (1.12-1.95) | Per 1 SD in NPX |
| CCL2 | Bernardino et al., 2023 | aOR = 1.0 (0.99–1.0) | Per ng/mL |
| hsTNT | de Leuw et al., 2021 | HR = 3.44 (1.31-9.07) | Per log_10_ |
| Total bilirubin | Marconi et al., 2018 | aHR = 0.74 (0.65-0.84) | Highest vs lowest quartile^‡^ |
| BNP | Reinsch et al., 2019 | aHR = 1.16 (1.01 to 1.33) | Per log_2_ |
|  |  |  |  |

^†^ Effect sizes are based on post-antiretroviral therapy baseline measurements for comparability between cohorts.

^‡^ No continuous measure was available.

Abbreviations: BNP, B-type Natriuretic Peptide; CCL2, Chemokine Ligand 2; CCL25, Chemokine Ligand 25; CI, Confidence Interval; Gal-9, Galectin-9; hsTNT / TNT, Troponin T (High-Sensitivity); IL-1Ra, IL-1 Receptor Antagonist; IL-1R1, IL-1 Receptor 1; IP-10, Interferon Gamma-Induced Protein 10; ITGA11, Integrin Subunit Alpha 11; NPX, Normalised Protein eXpression; PAI-1, Plasminogen Activator Inhibitor-1; PLA2G7, Phospholipase A2 Group VII; sCD, Soluble Cluster of Differentiation; sTNFR-I / sTNFR-II, Soluble TNF Receptors I and II; SD, Standard Deviation; suPAR, Soluble Urokinase-Type Plasminogen Activator Receptor.
