# Supplementary material for: Inflammatory Biomarkers Predicting Major Adverse Cardiovascular Events in People Living With HIV: A Systematic Review and Meta‐Analysis
Source: J Int AIDS Soc. 2026 Apr 27;29(4):e70101. doi: 10.1002/jia2.70101 (PMC13113420; doi:10.1002/jia2.70101)
Supplement: Supplementary file 5 — Supporting File 1: jia270101‐sup‐0005‐SupInfo‐File‐1.docx [file JIA2-29-e70101-s005.docx]

**Supporting Information File 1: “Inflammatory biomarkers predicting cardiovascular events in people living with HIV: a systematic review and meta-analysis”**

**Contents:**

1. Search strategy
2. Conversion of log-transformed biomarker scales

**1. Search strategy**

**Medline (Ovid MEDLINE® Epub Ahead of Print, In-Process & Other Non-Indexed Citations, Ovid MEDLINE® Daily and Ovid MEDLINE®) 1946 to present**

| **#** | **Query** | **Results from 01 May 2024** |
| --- | --- | --- |
| 1 | exp HIV/ or HIV.ab,ti. | 376944 |
| 2 | HIV infection*.ab,ti,kf. or exp HIV Infections/ | 338376 |
| 3 | AIDS serodiagnosis.ab,ti. or exp AIDS Serodiagnosis/ | 6751 |
| 4 | (PLWH or human immunodeficiency virus or acquired immunodeficiency syndrome).ab,ti,kf. | 115777 |
| 5 | (inflamm* or inflammatory response or pro-inflamm*).ab,ti. or exp Inflammation/ | 1480250 |
| 6 | (biomarker* or marker* or immune marker*).ab,ti. or exp Biomarkers/ | 1814936 |
| 7 | (cytokine* or CD antigen* or interleukin* or IL-6 or TNF or tumor necrosis factor or tumour necrosis factor).ab,ti,kf. or exp Cytokines/ | 1134440 |
| 8 | (C-reactive protein or CRP or hsCRP).ab,ti. or exp C-Reactive Protein/ | 125259 |
| 9 | d-dimer.ab,ti,kf. or exp Fibrin Fibrinogen Degradation Products/ | 21299 |
| 10 | (VCAM or ICAM).ab,ti. or exp Cell Adhesion Molecules/ | 161506 |
| 11 | (Brain Natriuretic Peptide or BNP or NTproBNP or NT-proBNP).ab,ti. or exp Natriuretic Peptide, Brain/ | 29494 |
| 12 | (galectin-3 or Gal-3).ab,ti. or exp Galectins/ | 9496 |
| 13 | (oxidi#ed low density lipoprotein or oxidi#ed LDL or oxLDL or myeloperoxidase).ab,ti. or exp Peroxidase/ | 47904 |
| 14 | troponin*.ab,ti. or exp Troponin/ | 37633 |
| 15 | matrix metalloproteinases.ab,ti. or exp Matrix Metalloproteinases/ | 62387 |
| 16 | (cardiovascular disease* or cardiovascular or cardiovascular event* or major adverse cardiovascular event* or vascular disease* or isch#em* or myocardial infarct* or acute coronary syndrome* or angina or peripheral arter* disease or sudden cardiac death or cardiac arrest or cardiovascular mortality or heart failure).ab,ti. or exp Myocardial Ischemia/ or exp Heart Arrest or exp Heart Failure/ or Peripheral Vascular Diseases/ | 1377663 |
| 17 | atherosclerosis.ab,ti. or exp Atherosclerosis/ | 157587 |
| 18 | (Percutaneous Coronary Intervention or revasculari#ation).ab,ti. or exp Percutaneous Coronary Intervention/ | 132158 |
| 19 | exp Stroke/ or (stroke or CVA or cerebrovascular accident).ab,ti. | 367288 |
| 20 | (Transient Ischemic Attack or TIA).ab,ti. or exp Ischemic Attack, Transient/ | 33020 |
| 21 | exp Risk/ or risk.tw. or exp Cohort Studies/ or cohort.tw. or exp Prognosis/ or "prognos*".tw. or "predict*".tw. or exp Incidence/ or incidence.tw. or exp Survival Analysis/ or survival.tw. or "causal factor".tw. or course.tw. or associat*.tw. | 12145648 |
| 22 | 1 or 2 or 3 or 4 | 463336 |
| 23 | 5 or 6 or 7 or 8 or 9 or 10 or 11 or 12 or 13 or 14 or 15 | 3833864 |
| 24 | 16 or 17 or 18 or 19 or 20 | 1759938 |
| 25 | 22 and 23 and 24 | 2781 |
| 26 | 21 and 25 | 2419 |

**Embase 1974 to present**

| **#** | **Query** | **Results from 01 May 2024** |
| --- | --- | --- |
| 1 | HIV.ab,ti. or exp *Human immunodeficiency virus/ | 478479 |
| 2 | HIV infection*.ab,ti,kf. or *Human immunodeficiency virus infection/ | 248866 |
| 3 | (acquired immunodeficiency syndrome or AIDS serodiagnosis).ab,ti. or exp *acquired immune deficiency syndrome/ | 307970 |
| 4 | PLWH.ab,ti,kf. or exp *Human immunodeficiency virus infected patient/ | 25598 |
| 5 | (inflamm* or inflammatory response or pro-inflamm*).ab,ti. or *chronic inflammation/ or *cardiovascular inflammation/ | 1815844 |
| 6 | (biomarker* or marker* or immune marker*).ab,ti. or exp biological marker/ | 1967627 |
| 7 | (cytokine* or CD antigen* or interleukin* or IL-6 or TNF or tumor necrosis factor or tumour necrosis factor).ab,ti,kf. or *cytokine/ | 1087099 |
| 8 | (C-reactive protein or CRP or hsCRP).ab,ti. or exp C reactive protein/ | 331889 |
| 9 | d-dimer.ab,ti,kf. or exp D dimer/ | 48913 |
| 10 | (VCAM or ICAM).ab,ti. or exp cell adhesion molecule/ | 73430 |
| 11 | (Brain Natriuretic Peptide or BNP or NTproBNP or NT-proBNP).ab,ti. or exp brain natriuretic peptide/ | 74922 |
| 12 | (galectin-3 or Gal-3).ab,ti. or exp galectin/ | 11194 |
| 13 | (oxidi#ed low density lipoprotein or oxidi#ed LDL or oxLDL or myeloperoxidase).ab,ti. or exp oxidized low density lipoprotein/ or exp myeloperoxidase/ | 66138 |
| 14 | troponin*.ab,ti. or exp troponin/ | 97064 |
| 15 | matrix metalloproteinases.ab,ti. or exp matrix metalloproteinase/ | 48788 |
| 16 | (cardiovascular disease* or cardiovascular event* or major adverse cardiovascular event* or vascular disease* or isch#em* or myocardial infarct* or acute coronary syndrome* or angina or peripheral arter* disease or sudden cardiac death or cardiac arrest or cardiovascular mortality or heart failure).ab,ti. or exp heart death/ or exp heart failure/ or exp ischemic heart disease/ or exp major adverse cardiac event/ or exp *aneurysm/ or exp coronary artery disease/ or exp *peripheral vascular disease/ or exp sudden cardiac death/ | 2689587 |
| 17 | atherosclerosis.ab,ti. or exp atherosclerosis/ | 326847 |
| 18 | (Percutaneous Coronary Intervention or revasculari#ation).ab,ti. or exp *percutaneous coronary intervention/ | 176277 |
| 19 | (stroke or CVA or cerebrovascular accident).ab,ti. or exp cerebrovascular accident/ | 609306 |
| 20 | (Transient Ischemic Attack or TIA).ab,ti. or exp transient ischemic attack/ | 62641 |
| 21 | exp risk/ or risk.tw. or exp cohort analysis/ or cohort.tw. or exp prognosis/ or "prognos*".tw. or "predict*".tw. or exp Incidence/ or incidence.tw. or exp survival analysis/ or survival.tw. or "causal factor".tw. or course.tw. or associat*.tw. | 15050907 |
| 22 | 1 or 2 or 3 or 4 | 732166 |
| 23 | 5 or 6 or 7 or 8 or 9 or 10 or 11 or 12 or 13 or 14 or 15 | 4330608 |
| 24 | 16 or 17 or 18 or 19 or 20 | 3026087 |
| 25 | 22 and 23 and 24 | 5429 |
| 26 | 25 and 21 | 4482 |

**2. Conversion of log-transformed biomarker scales**

**Rationale**

To ensure comparability across studies reporting effect estimates, such as odds ratios (ORs) or hazard ratios (HRs) on different scales, we applied a standardised conversion approach where feasible. Included studies reported biomarker levels using a variety of transformations. The most common were log-transformed scales, typically log base 10 (log₁₀) or log base 2 (log₂), although some studies reported biomarkers on a continuous linear scale without transformation, or categorised values into quantiles such as quartiles.

For transparency and reproducibility, we maintained two versions of the data extraction spreadsheet. The first contained the effect estimates as originally reported in the studies. The second contained any values that had been transformed or rescaled to ensure comparability across studies.

With respect to extraction, continuous measures were preferred to categorical measures in order to avoid bias from categorisation and support cross-study comparability.

**Conversion between log-transformed scales**

The usual interpretation of an OR or a HR for a continuous biomarker is the relative change in risk per one-unit increase in the biomarker on the scale being used. When the biomarker is transformed using a logarithmic scale, the unit increase corresponds to a multiplicative change in the original (untransformed) biomarker concentration. For example, on a log₁₀ scale, a one-unit increase in the log-transformed biomarker corresponds to a 10-fold increase in the original concentration. This follows directly from:

$$\log_{10}(1)=0$$

$$\log_{10} \left( 10 \right)=1$$

If we look at a biomarker reported on the natural logarithm scale, or log*ₑ* scale, a one-unit increase corresponds to an e-fold increase in the biomarker concentration.

$$\log_{e}\left( 1 \right)=0$$

$$\log_{e}\left( 10 \right)=2.302585$$

To convert ORs or HRs reported per log*ₑ* unit increase to an equivalent per log₁₀ unit increase, we applied the following transformation:

$${OR}_{\log_{10}}=\left( {OR}_{\log_{e}} \right)^{2.302585}$$

To solve for ${OR}_{\log_{e}}$, we raised both sides of the equation to the power of 1/2.302585 to yield:

$${OR}_{\log_{e}}=\left( {OR}_{\log_{log}} \right)^{0.434294}$$

The same rescaling approach applies to the corresponding confidence intervals. This is purely a mathematical conversion between scales and does not require additional assumptions.

**Studies reporting effect estimates per 1 standard deviation (SD)**

Some studies reported effect estimates per one standard deviation (SD) increase in biomarker concentration following log transformation. To allow comparability with other studies that reported effect estimates per 1 log₁₀ unit increase, we rescaled these estimates using the reported SD of the biomarker in the study population. Specifically, we converted the effect size from per 1 SD to per 1 log₁₀ unit using the following formula:

$$OR_{\log_{10}}=\left( {OR}_{per 1 SD} \right)^{{1/SD}_{\log_{10}}}$$

**Continuous, untransformed scales**

For studies that reported effect estimates based on continuous, untransformed biomarker concentrations, direct comparison with studies using log-transformed measures would require access to individual participant data from the study. In the absence of such data, we did not attempt to convert these results and therefore did not include them in meta-analyses alongside studies reporting log-transformed outcomes.
